# Global transcriptome analysis and characterization of *Dryopteris fragrans* (L.) Schott sporangium in different developmental stages

**DOI:** 10.1186/s12864-018-4843-2

**Published:** 2018-06-18

**Authors:** Zhen Lu, Qingyang Huang, Tong Zhang, Baozhong Hu, Ying Chang

**Affiliations:** 1 Laboratory of Plant Research, College of Life Sciences, Northeast Agricultural University, Harbin, 150030 China; 2 Harbin University, Harbin, 150086 China; 3 Institute of Natural Resources and Ecology, Heilongjiang Academy of Sciences, Harbin, 150040 China

**Keywords:** Transcriptome, *Dryopteris fragrans*, Sporangia, Development

## Abstract

**Background:**

*Dryopteris fragrans* (*D. fragrans*) is a potential medicinal fern distributed in volcanic magmatic rock areas under tough environmental condition. Sporangia are important organs for fern reproduction. This study was designed to characterize the transcriptome characteristics of the wild *D. fragrans* sporangia in three stages (stage A, B, and C) with the aim of uncovering its molecular mechanism of growth and development.

**Results:**

Using a HiSeq 4000, 79.81 Gb clean data (each sample is at least 7.95 GB) were obtained from nine samples, with three being supplied from each period, and assembled into 94,705 Unigenes, among which 44,006 Unigenes were annotated against public protein databases (NR, Swiss-Prot, KEGG, COG, KOG, GO, eggNOG and Pfam). Furthermore, we observed 7126 differentially expressed genes (DEG) (Fold Change > 4, FDR < 0.001), 349,885 SNP loci, and 10,584 SSRs.

DEGs involved in DNA replication and homologous recombination were strongly expressed in stage A, and several DEGs involved in cutin, suberin and wax biosynthesis had undergone dramatic changes during development, which was consistent with morphological observations. DEGs responsible for secondary metabolism and plant hormone signal transduction changed clearly in the last two stages.

DEGs homologous to those known genes associated with the development of reproductive organs of flowering plants have also been validated and discussed, such as AGL61, AGL62, ONAC010. In particular, a Unigene encoding TFL1, an important flower–development regulator in flowering plants, was identified and exhibited the highest expression level in stage B in *D. fragrans* sporangia.

**Conclusions:**

This study is the first report on global transcriptome analysis in the development of sporangia of wild *D. fragrans*. DEGs related to development and homologous to flower-seed development in flowering plants were discussed. All DEGs involved in DNA replication and homologous recombination were consistent with morphological observations of paraffin slices. The results of this study provide rare resources for further investigation of the *D. fragrans* sporangium development, stress resistance and secondary metabolism.

**Electronic supplementary material:**

The online version of this article (10.1186/s12864-018-4843-2) contains supplementary material, which is available to authorized users.

## Background

The sporangium of ferns, the place that produces spores, plays an important role in the propagation of the plants, similar to that of the flower and fruit of flowering plants. At present, a number of investigations of the morphological development, evolution and functions of the sporangium have been reported [1–4], but little is known regarding the transcriptome of fern sporangium.

*D. fragrans* (L.) Schott, also known as lava grass in China, is a perennial fern in the *Dryopteris* genus of the *Dryopteris* family. This species belongs to the single Fragrantes clade of a sister to the rest of *Dryopteris* in a molecular circumscription study [5]. Leaves and sori have dense glandular trichomes. The plants have a slight fragrance and are hence called *D. fragrans*.

*D. fragrans* is primarily distributed in Asia including China, Japan, Korea and Russia. In China, this species mainly grows in Heilongjiang, Jilin and Liaoning Provinces. This species could survive under harsh natural environmental conditions, such as uncovered volcanic magmatic rock areas. For instance, in Wudalianchi, Heilongjiang Province, one of the most prosperous places for *D. fragrans* in China, this grass grows on the lava rock where lava flowed hundreds of years ago without any shade. There are insect stressors because the habitat is next to farmlands, lakes, copses and orchards with fertile soil from the molten lava, and the lowest and highest temperature were − 35 °C and 35 °C, respectively, in 2016.

Different from other types of ferns, wild *D. fragrans* faces a variety of biotic and abiotic stressors during sporangia development, which provides a unique resource for investigating both the development and the stress resistance of sporangia.

In this study, we collected three periods of sporangia from those *D. fragrans* growing on the lava near Bagua Lake in Wudalianchi in July 2016. A transcriptome database was established using HiSeq 4000, and it will further our understanding of the development of fern sporangia with natural stress responses.

## Methods

*D. fragrans* sporangia (include sporangiophore and indusium) were collected from the lava rock near Bagua Lake, Wudalianchi, Heilongjiang, China located at 48.733334 N, 126.167966E as the center with a radius of 30 m between July 1, 2016 and July 6, 2016 under permission from the government.

**Fig. 1:**
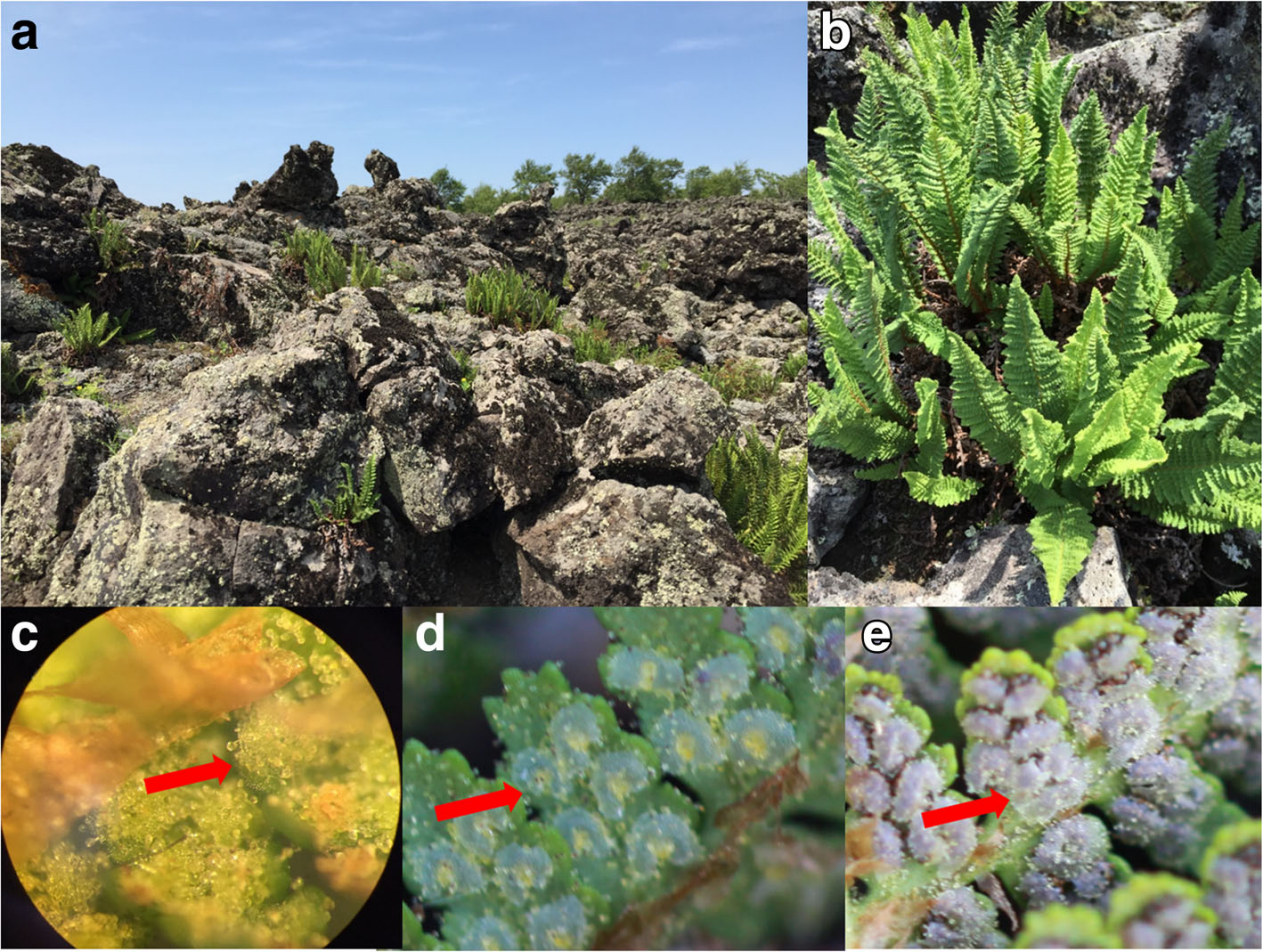
**a**. Natural habitat of *D. fragrans* (the sampling site) **b**. *D. fragrans* **c**.,**d**.,**e**. Stage A, B and C. Arrow for sporangium, golden highlights on sporangium are glandular trichomes

In our research, *D. fragrans* sporangia were divided into three stages, A, B and C, according to their obviously different morphology. Sporangia in Stage A were light green and visible to the naked eye on spore leaves fully expanded. Those in Stage B were pale green and extended to the edge of the leaf blade, containing sand-like particles. The hoar sporangia with mature spores were assigned to Stage C (Fig. 1).

To ensure the uniformity of samples, only those having at least 15 leaves, including circinate leaves and covering all of the three stages of sporangia plants, were chosen. The sporangia were kept in 1.5 ml tubes in liquid nitrogen immediately after being removed from the plant. All of the samples brought back to Northeast Agricultural University were stored in liquid nitrogen before use.

For the transcriptome, we did three biological repeats at each stage (Stage A: T01, T04, T07; Stage B: T02, T05, T08; Stage C: T03, T06, T09). In the qRT-PCR assay, three biological replicates with three technique repeats were performed using the same samples for RNAseq.

Paraffin section samples were collected from Wudalianchi, fixed with formalin/acetic acid/alcohol (FAA) immediately after collection, and later brought back to the laboratory for follow-up treatment.

The optical microscope pictures were photographed in the laboratory, and the samples were transplanted from the field.

### RNA quantification and qualification

Total RNA was extracted with a TIANGEN RNAprep Pure Plant Kit.

The purity, concentration and integrity of RNA samples were determined by a Nanodrop 2.0 and Aglient 2100 to ensure the use of qualified samples for transcriptome sequencing.

### Library preparation for transcriptome sequencing

The library was constructed by enrichment of eukaryotic mRNAs with magnetic beads with Oligo(dT), and mRNA was randomly interrupted by adding Fragmentation Buffer. The first cDNA chain was synthesized using mRNA as the template and random hexamers as a six-base random primer. Next, the first cDNA chain was added to the buffer solution. The second cDNA chain was synthesized by RNase H and DNA polymerase I, and cDNA was purified by AMPure XP beads. The purified double-stranded cDNA was followed by terminal repair followed by the addition of a tail and the attachment of the sequenced connector. Next, AMPure XP beads were used to select the fragment size. Finally, the cDNA library was obtained by PCR enrichment.

After the completion of the library construction, we used a Qubit 2.0 and Agilent 2100 to detect the library’s concentration and the size of the inserted fragment (Insert Size). The effective concentration of the library was accurately quantified with Q-PCR. After being qualified, an HiSeq 4000 was used for high-throughput sequencing.

### Transcriptome assembly and gene annotation

After obtaining high quality sequencing data, the sequence was assembled by Trinity [6].

The Unigene sequence was compared with NR, Swiss-Prot, GO, COG, KOG, eggNOG4.5 and KEGG databases using BLAST software. Using KOBAS2.0 to get the KEGG Orthology results from Unigene in KEGG. The Unigene amino acid sequence was predicted and compared with the Pfam database by HMMER software to obtain the annotated Unigene information.

The relevant software and methods for gene structure analysis such as Open Reading Frames (ORFs), Simple Sequence Repeats (SSRs) and Single Nucleotide Polymorphisms (SNPs) are shown in Additional File 1.

## Results

### Sequence analysis and assembly

To obtain a comprehensive overview of the sporangia of *D. fragrans* (L.) Schott, we used high-throughput sequencing by HiSeq4000. After quality control, we obtained 79.81 GB clean data, and the percentage of Q30 in different simples are not less than 90.72%. The read number (pair-end reads from clean data) of each sample ranges from 25,642,892 to 34,674,344, and all GC contents were between 48.48 and 48.06% (Table 1).

**Table 1:** Summary of transcriptomes from sporangia

ID	Stage	Read Number	Base Number	GC Content	% ≥ Q30
T01	A	26,342,364	7,841,967,940	47.10%	91.94%
T02	B	29,806,735	8,839,906,000	46.48%	92.05%
T03	C	31,621,236	9,378,990,718	47.81%	91.88%
T04	A	25,642,982	7,591,661,296	47.24%	92.08%
T05	B	34,138,269	10,141,040,054	46.66%	90.72%
T06	C	30,367,322	9,004,606,578	48.06%	92.43%
T07	A	34,674,344	10,293,447,866	47.14%	91.01%
T08	B	25,984,662	7,717,620,272	46.60%	91.78%
T09	C	30,267,355	8,999,977,228	47.84%	91.70%

**Fig. 2:**
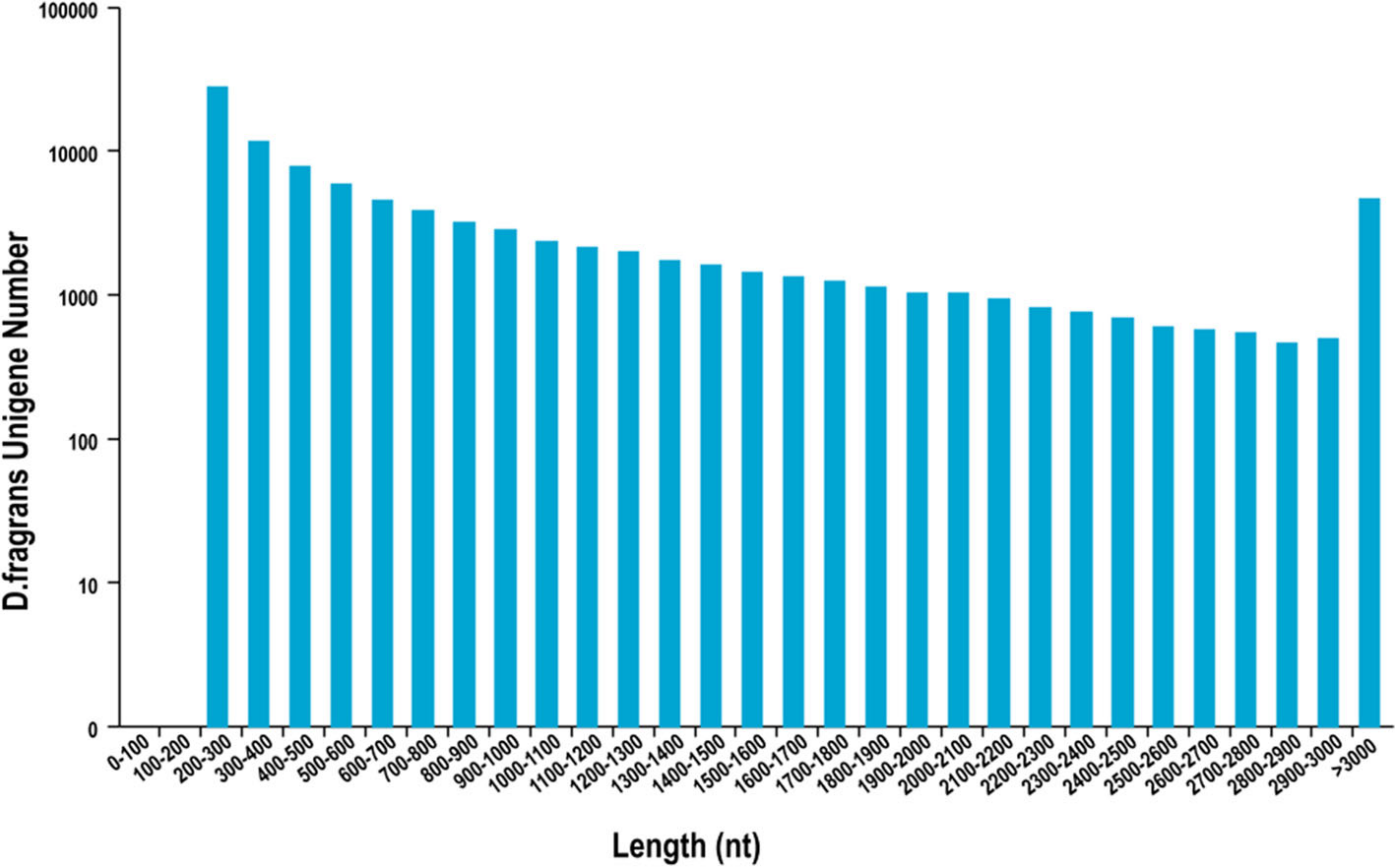
*D.fragrans* Unigene Length Distribution

**Fig. 3:**
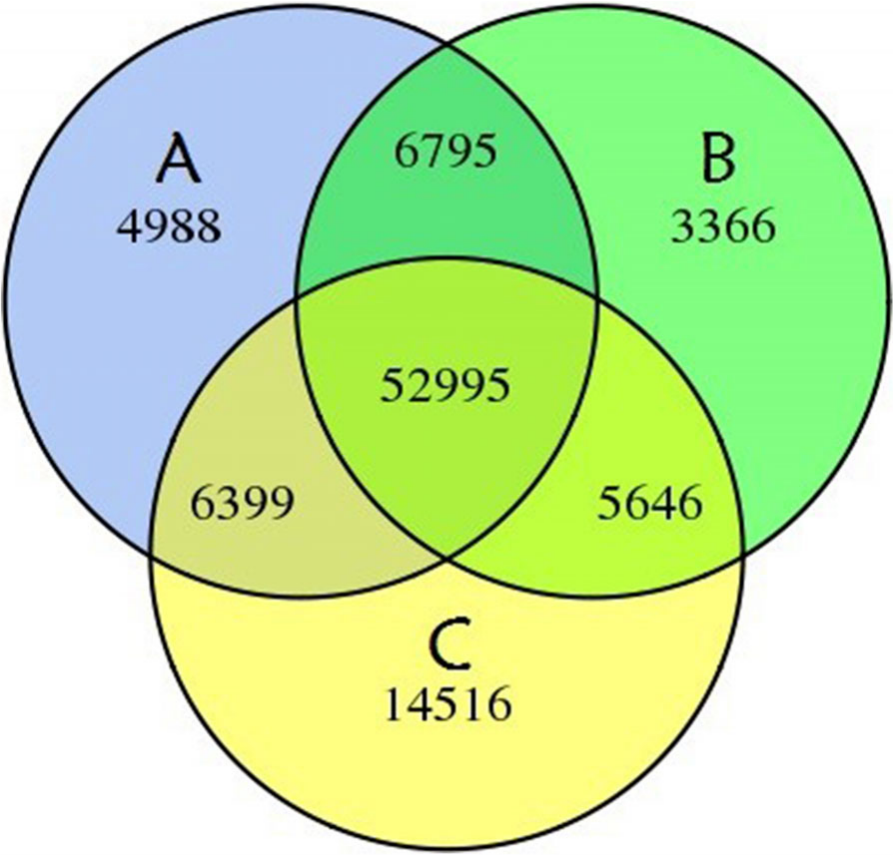
Venn diagram shows the number of expressed genes in the A, B and C stage of sporangia

A total of 94,705 Unigenes with a mean length of 913.87 bp and an N50 of 1631 bp (50% of the assembled bases were equal or greater than 1631 bp). The length distribution of sporangia of *D. fragrans* is shown in Fig. 2 and Table 2, with 28.80% of all Unigenes showing lengths longer than 1 kb. A Venn diagram of the expressed Unigenes is shown in Fig. 3. A total of 52,995 Unigenes were expressed in three periods, and the numbers of genes specifically expressed in stage A, B and C were 4988, 3366 and 14,516, respectively. All reads generated in this study are shown in Additional file 1.

**Table 2:** Assembly results statistics

Length Range	Transcript	Unigene
200–300	31,911(18.62%)	27,780(29.33%)
300–500	27,838(16.24%)	19,474(20.56%)
500–1000	37,142(21.67%)	20,176(21.30%)
1000–2000	40,294(23.51%)	15,814(16.70%)
2000+	34,188(19.95%)	11,461(12.10%)
Total Number	171,373	94,705
Total Length	209,925,678	86,547,872
N50 Length	2021	1631
Mean Length	1224.96	913.87

Percentage representation in parentheses indicates the proportion of Unigene in the corresponding length rage. N50 Length: Represents the length of the N50 of the Unigene; Mean Length: Represents the average length of Unigene

### Simple sequence repeat (SSR) and single nucleotide polymorphism (SNP) analysis

**Fig. 4:**
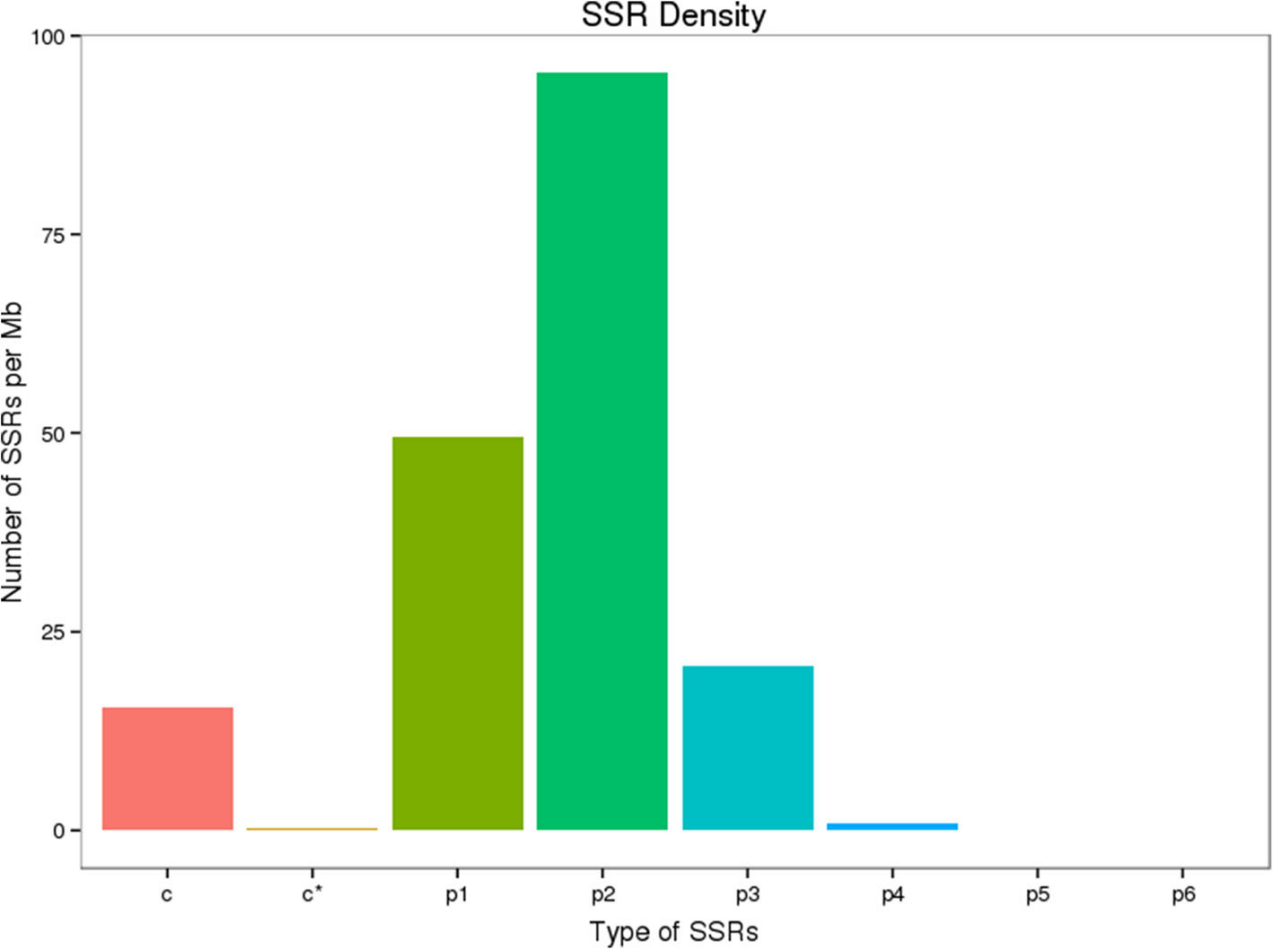
C (Mixed SSR,contains at least two perfect SSR, and the distance is less than 100 bp), c* (Mixed SSR with more than two times repeat), p1 (Mono-nucleotides), p2 (Di-nucleotides), p3 (Tri-nucleotides), p4 (Tetra-nucleotides), p5 (Penta-nucleotides), p6 (Hexa-nucleotides).

**Fig. 5:**
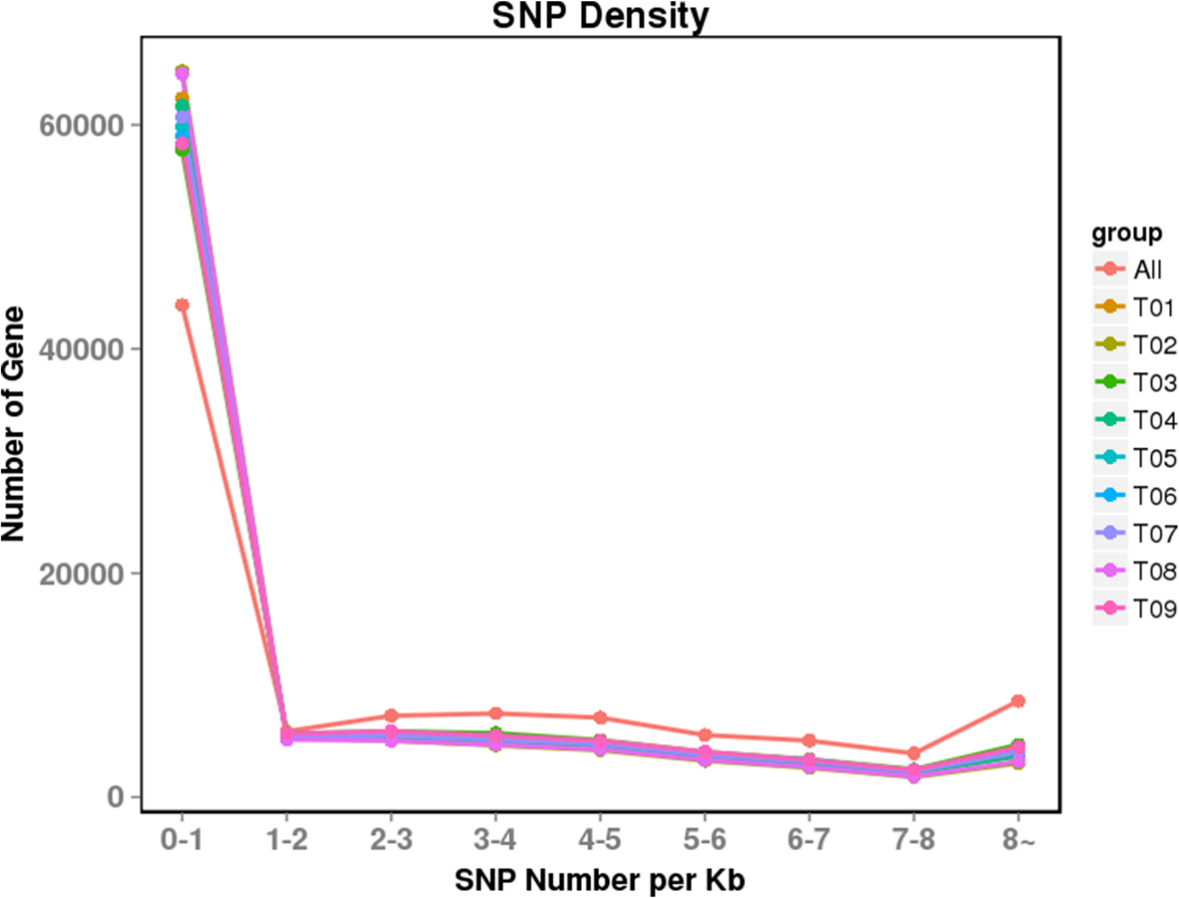
The SNP density distribution map. The abscissa is SNP density, that is, the number of SNP per Kb gene sequence; the ordinate is the number of genes that have the corresponding density

SSR analysis was based on Unigenes longer than 1 kb, and 10,584 SSRs were identified in 27,275 Unigenes (Unit size/minimum number repeats were setting (1/10) (2/6) (3/5) (4/5) (5/5) (6/5) and interruptions of max difference for 2 SSRs is 100). The most abundant repeat motifs were di-nucleotides (5541, 52.35%) followed by mono-nucleotides (2874, 27.15%) and tri-nucleotides (1197, 11.30%) (Fig. 4). By comparing the reads and Unigene sequences of the nine samples, a total of 349,885 single nucleotide polymorphisms (SNPs) were determined. The density statistics of SNPs in Unigenes indicated that most Unigenes (43,907, 46.36%) had 0–1 SNPs per Kb, and 8589 (9.07%) Unigenes had SNP density over 8 per Kb (Fig. 5).

### Global analysis showing the difference between the three stages of sporangia

**Fig. 6:**
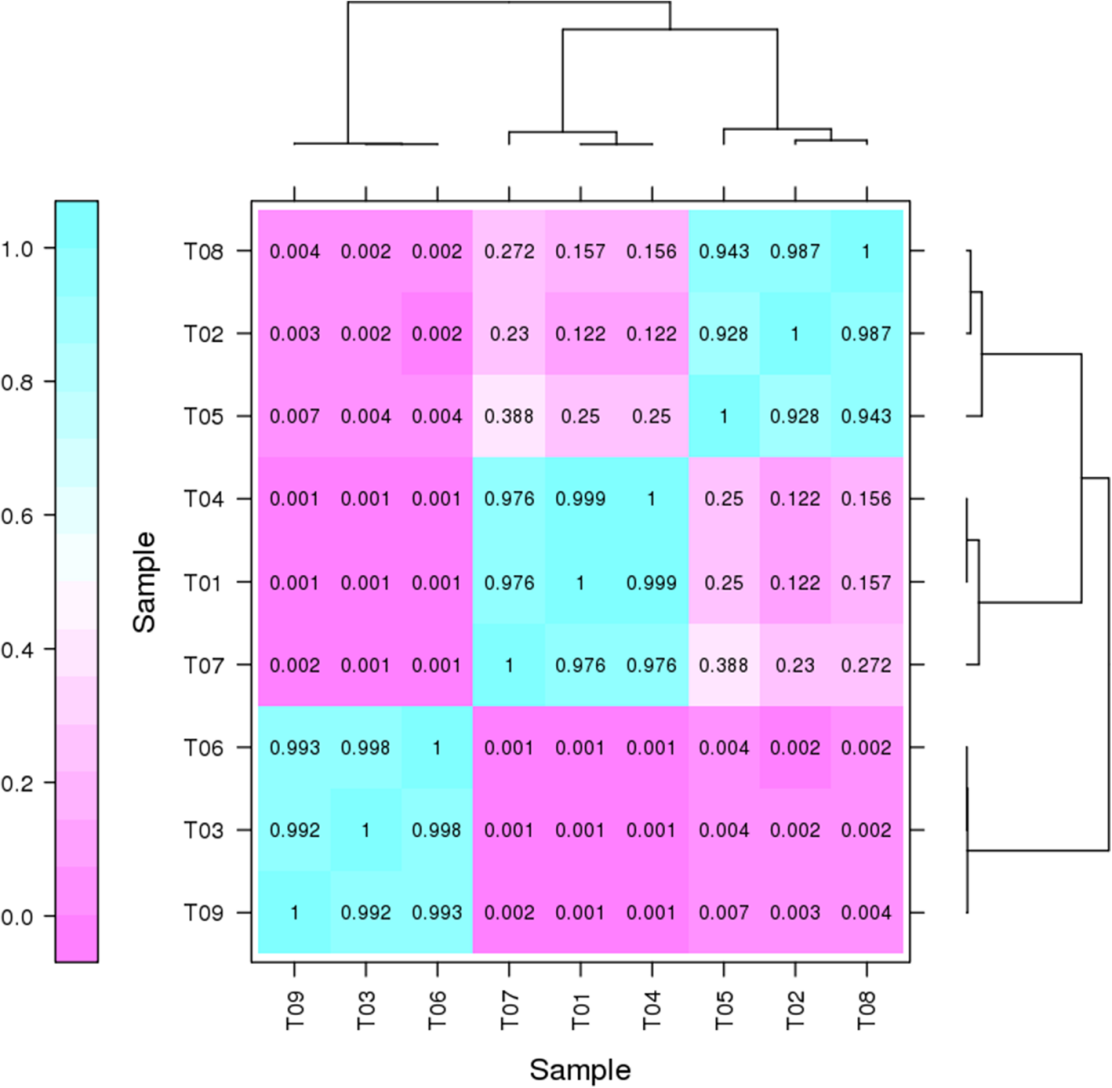
The correlation of different samples

To understand the difference in samples of biological repetitions and between each stage, 36 scatter diagrams of gene pairwise correlations were drawn (Additional file 2), and a cluster was drawn showing the biological repetition correlations (Fig. 6). The correlation coefficients among biological repetitions showed high correlations, and the differences between stages were evident. These data indicate that the sporangia of these three periods were obviously different at the transcriptional level.

### Functional annotation and classification

In all, 44,006 of the 94,705 Unigenes were homologous with sequences in public protein databases (NR, Swiss-Prot, KEGG, COG, KOG, GO, eggNOG and Pfam) with a cut-off E-value of 10e-5. A total of 15,975 Unigenes matched in the COG Annotation, and 21,928, 14,913, 25,772, 31,677, 23,887, 40,008, 40,535, and 44,006 Unigenes matched in the GO Annotation, KEGG Annotation, KOG Annotation, Pfam Annotation, Swissprot Annotation, eggNOG Annotation, and nr Annotation, respectively (Table 3).

**Table 3:** Unigene annotation statistics

#Anno_Database	Annotated_Number	300 < =length < 1000	length > =1000
COG_Annotation	15,979	4389	8369
GO_Annotation	21,928	6476	10,823
KEGG_Annotation	14,913	4462	8034
KOG_Annotation	25,772	7952	13,524
Pfam_Annotation	31,677	9006	18,101
Swissprot_Annotation	23,887	6793	14,107
eggNOG_Annotation	40,008	12,015	20,080
nr_Annotation	40,535	12,076	20,616
All_Annotated	44,006	13,509	21,241

### GO classification

**Fig. 7:**
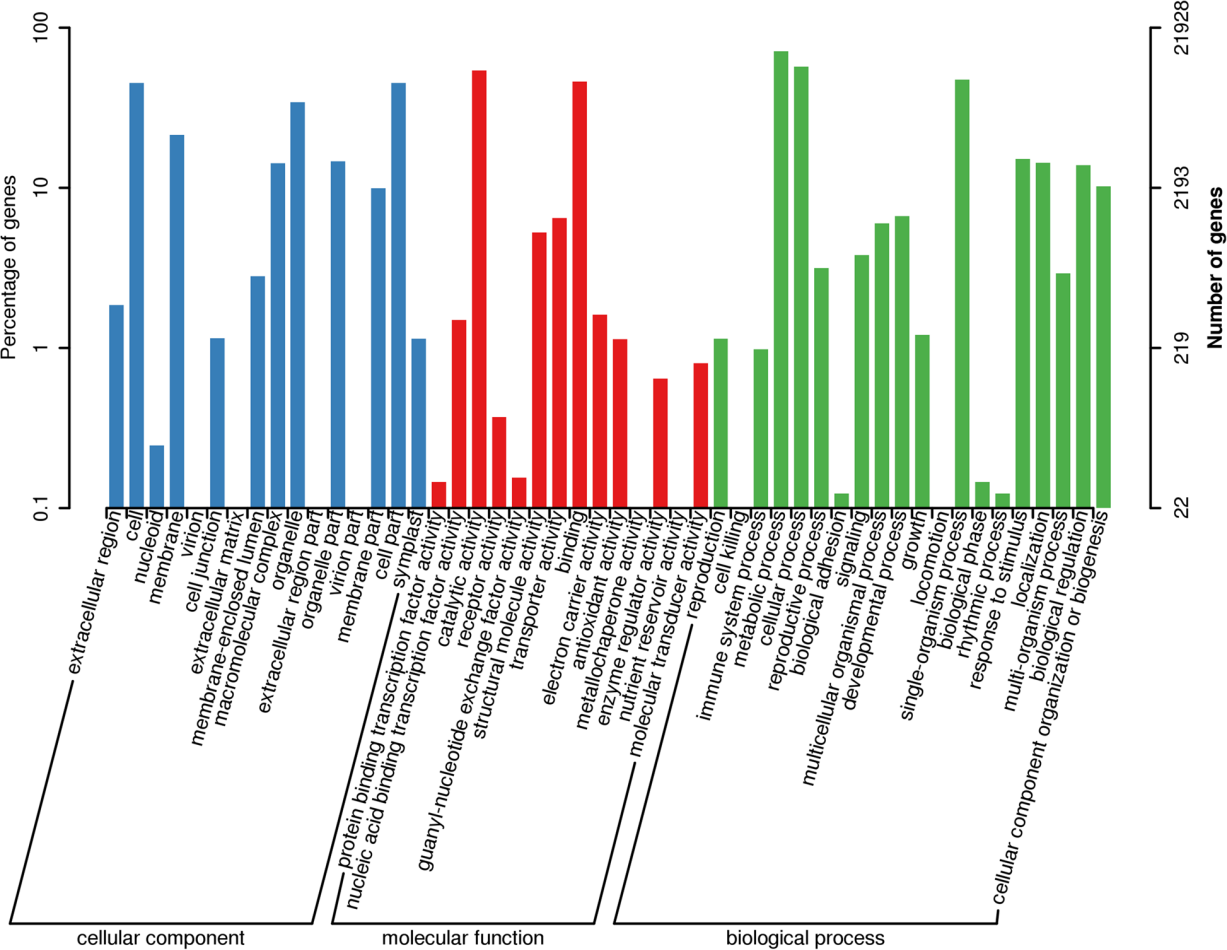
GO classification

The GO database is a structured standard biological annotation system that applies to all species. The database divides 21,928 matched Unigenes into multiple layers and the lower level node represented by more specific functions. “Cell part” (9879, 10.43%), “cell” (9879, 10.43%) and “organelle” (7520, 7.94%) had the highest number of matches in the “cellular component”. “Catalytic activity” (11,891, 12.56%) and “binding” (10,124, 10.69%) accounted for significantly higher than other categories in “molecular function.” In biological processes, “metabolic process” (15,628, 16.50%), “cellular process” (12,528, 13.23%) and “single-organism process” (10,367, 1095%) were most enriched (Fig. 7).

### eggNOG function class

**Fig. 8:**
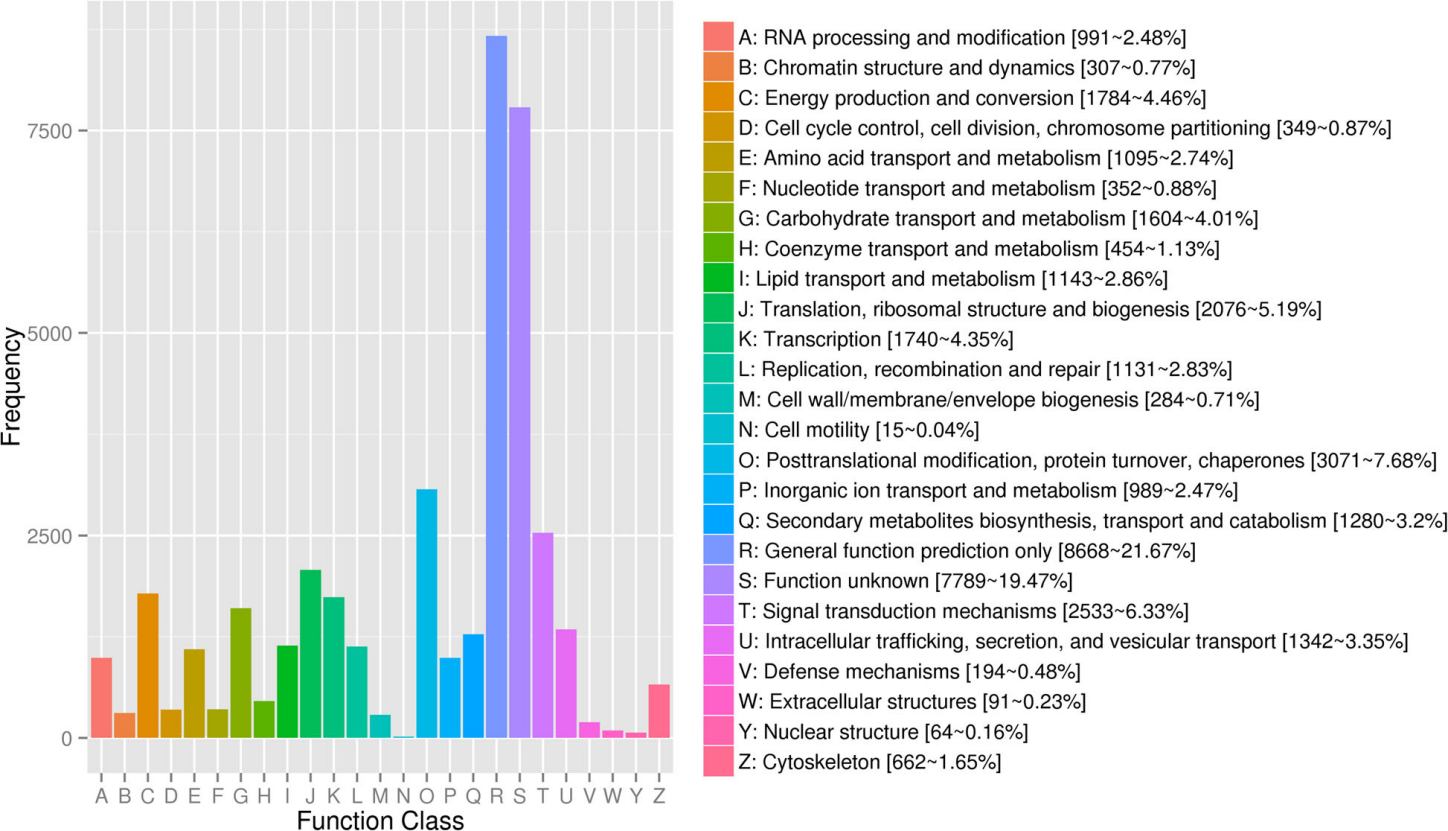
eggNOG Function Classification of Consensus Sequence.

The eggNOG (v4.5) database contains the functional description and functional classification of the orthologous proteins, including COG, KOG, and a number of other proteins. In this study, 40,008 Unigenes were allocated to 25 eggNOG classifications. Excluding the largest proportion of general function prediction only (8668, 21.67%) and function unknown (7789, 19.47%) classes, the top three classes were posttranslational modification, protein turnover, chaperones (3071, 7.68%), signal transduction mechanisms (2533, 6.33%), and translation, ribosomal structure and biogenesis (2076, 5.19%) (Fig. 8).

### Nr homologous species distribution

**Fig. 9:**
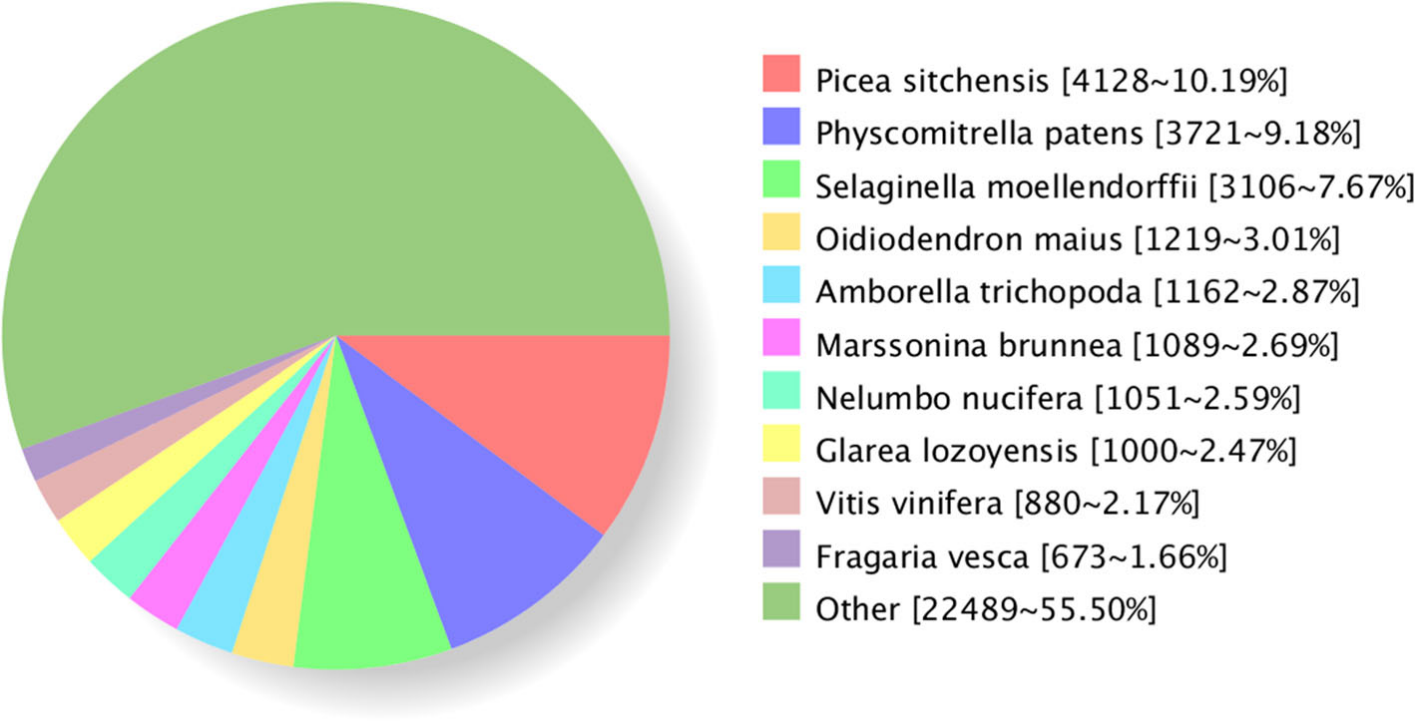
Nr Homologous Species Distribution

The outcome of the homology search of Unigenes in Nr databases showed 4128 (10.19%) Unigenes were homologous to sequences of *Picea sitchensis*, 3721 (9.18%) of which were mapped in *Physcomitrella patens*, and 3106 (7.67%) were *Selaginella moellendorffii*. Unigenes matched to the remaining species were less than 5% (Fig. 9).

### DEGs between different stages

**Fig. 10:**
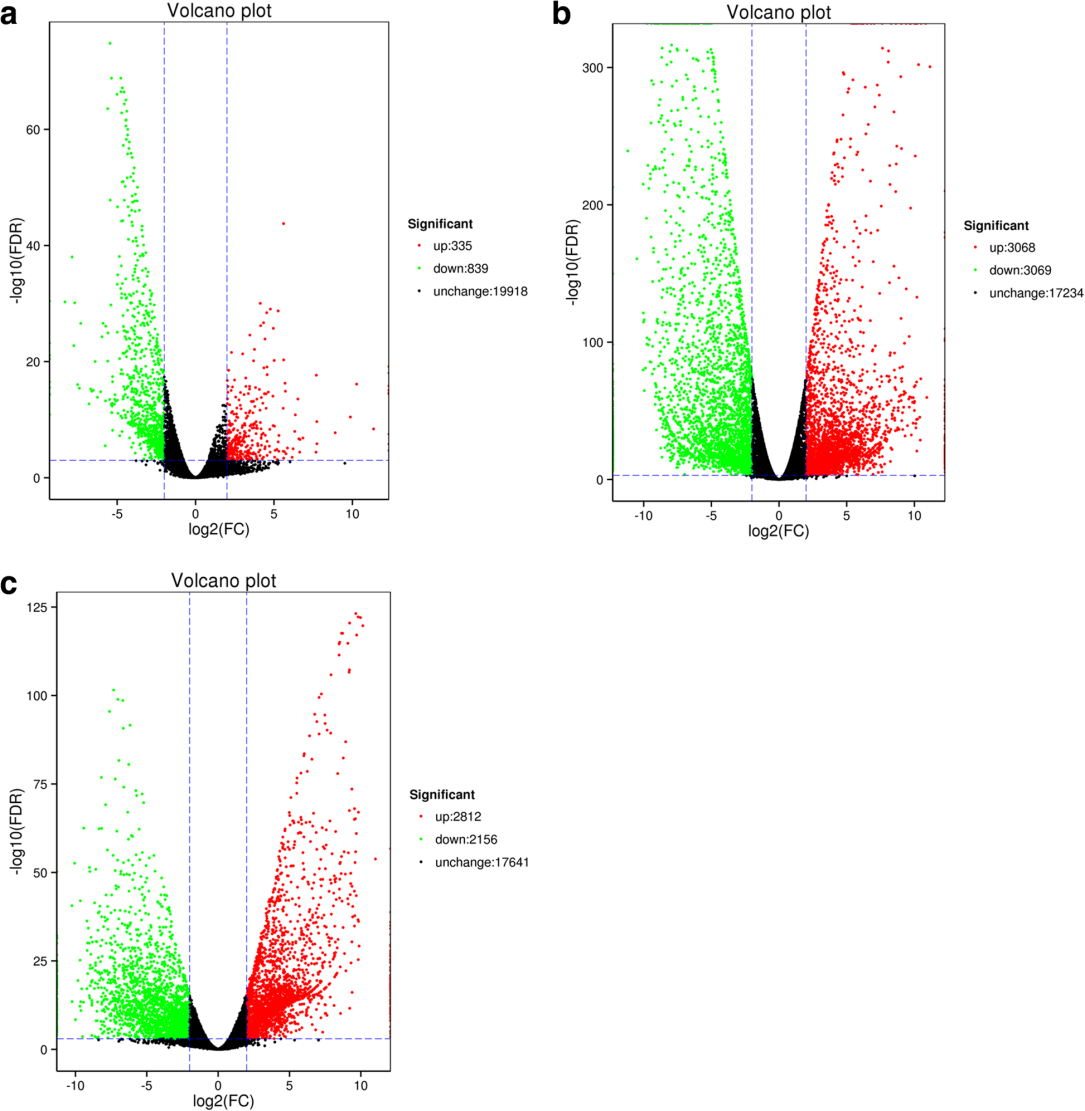
Volcanic map of differentially expressed gene **a**. stage A vs B **b**. stage A vs C **c**. stage B vs C. Each point represents a gene expression, the abscissa of a single gene in the two sample volume fold difference on the value, the vertical axis represents the false discovery rate of negative value. In green and red dots represent significant differences of gene expression, gene expression in green, red represents gene expression was up-regulated, the black points represent no significant differences of gene expression

DESeq was used for differential expression analyzing the sporangia of *D. fragrans* at different stages, and 7126 differentially expressed genes were obtained (Fold Change > 4, FDR < 0.001) (Additional file 3). AvsC had the biggest difference. There were 3068 up-regulated and 3069 down-regulated Unigenes in stage A compared with stage C (Fig. 10), and annotated DEGs were listed in Table 4.

**Table 4:** Annotated differential expression gene quantity statistics

#DEG_Set	Annotated	COG	GO	KEGG	KOG	Pfam	Swiss-Prot	eggNOG	nr
AvsB	660	204	287	207	317	524	412	584	631
AvsC	4164	1552	2124	1405	2148	3469	2803	3842	4043
BvsC	3397	1300	1746	1113	1781	2821	2223	3136	3302

### GO function enrichment of *D. fragrans* in different stages

**Fig. 11:**
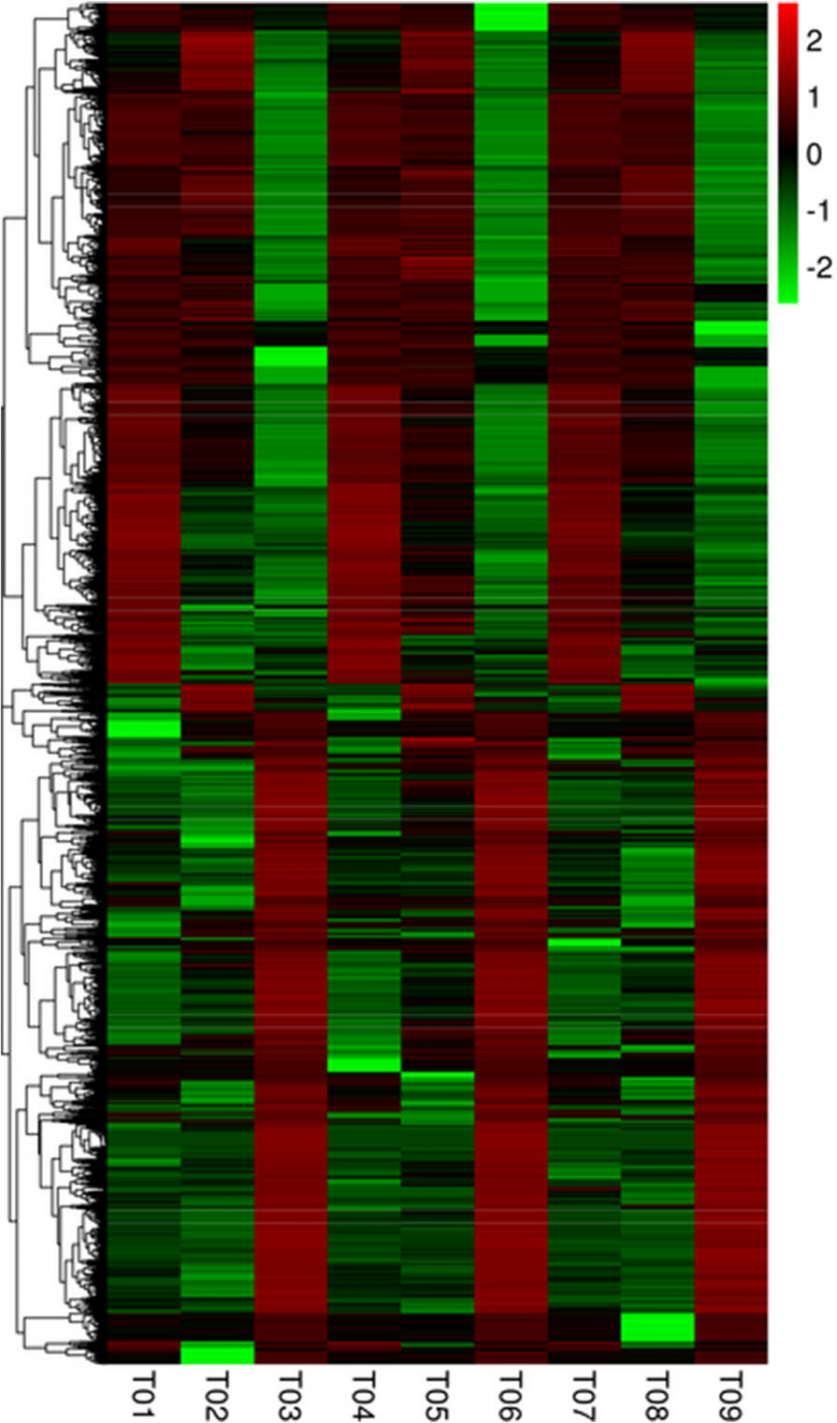
Heat map of all differentially expressed genes were clustered

We plotted the annotated statistical graph of differentially expressed genes GO two class nodes (Fig. 11). We performed hierarchical cluster analysis of all the DEGs and clustered them into a heatmap of enrichment of GO terms with 66 groups (Additional file 4).

The expression levels of most DEGs were highly similar among the biological replicates in each stage, and certain of them were composed of larger groups (e.g., group 4, group 9, group 10, and group 63), while others were smaller groups (e.g., group 18, group 31, group 36 and group 51). Some unigenes expression levels were not stable in three repeats in the same stage, but we think it was not caused by experimental errors because these unigenes can still be clustered according to GO terms (e.g., group1, group.34, and group 66).

### Enrichment analysis of DEGs of *D. fragrans* in different stages in the KEGG pathway

**Fig. 12:**
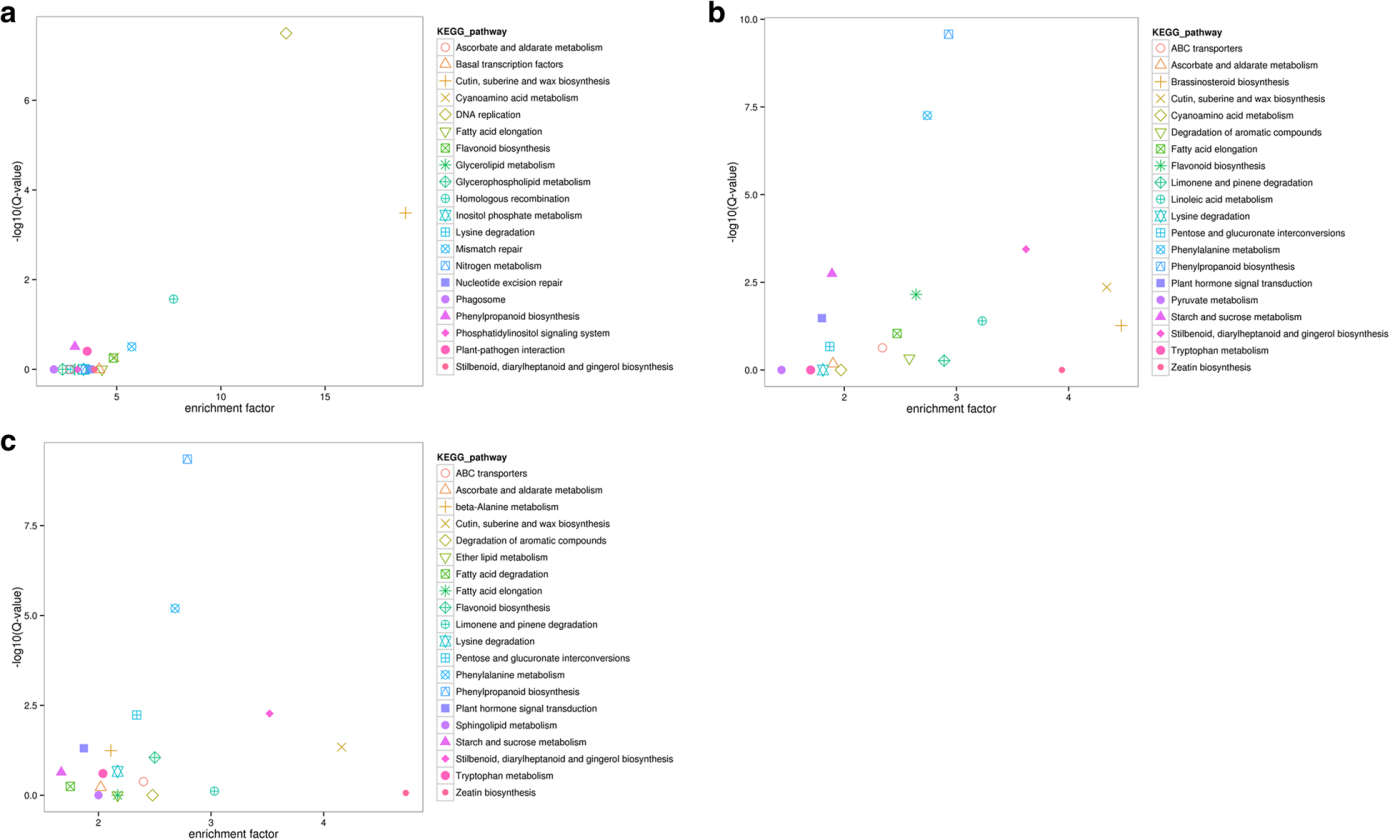
Differential gene expression of KEGG pathway enrichment scatter diagram. **a**. stage A vs B **b**. stage A vs C **c**. stage B vs C. The abscissa is the enrichment factor, the bigger the enrichment factor, the more significant the enrichment level of differentially expressed genes in the pathway. The ordinate is log10 (Q value), the greater the ordinate, the greater the significance of differentially expressed genes in the pathway

Using enrichment factor concentrations of the pathway, and after the calculation was significantly enriched by the Fisher exact test method, we obtained a KEGG enrichment sheet (Additional file 5), and the top 20 pathways with highest enrichment factor are displayed in the figure (Fig. 12). The pathways with most representation between stage A and B by the Unigenes were cutin, suberin and wax biosynthesis [ko00073] (enrichment factor 18.86), DNA replication [ko03030] (13.13), homologous recombination [ko03440] (7.73), and mismatch repair [ko03430](5.72). The highest enrichment factor between stage B and C were zeatin biosynthesis [ko00908](4.73), cutin, suberin and wax biosynthesis [ko00073] (4.16), stilbenoid, diarylheptanoid and gingerol biosynthesis [ko00945] (3.52) and brassinosteroid biosynthesis [ko00905](3.06). We observed that DNA replication [ko03030] (between stage A and B, 13.13, 3.21E-8), cutin, suberin and wax biosynthesis [ko00073] (between stage A and B,18.06, 0.000326) and homologous recombination [lo03440] (between stage A and B, 7.73, 0.026986) had enrichment factors > 5 with corrected *P*-values ≤0.05 in at least one set of data.

### Validation of DEGs by qRT-PCR

We selected 8 genes (c114077.graph_c0, c110186.graph_c0, c111664.graph_c0, c120866.graph_c0, c121145.graph_c1, c109839.graph_c1, c117958.graph_c0, c92612.graph_c0) (Additional file 6) for validation according to the results of KEGG pathway enrichment analysis (relationship between sporangia of *D. fragrans* development and flower development). The validation (Additional file 7) showed that the relative expression level and FPKM had the same trend (Additional file 8).

### Paraffin section observations

**Fig. 13:**
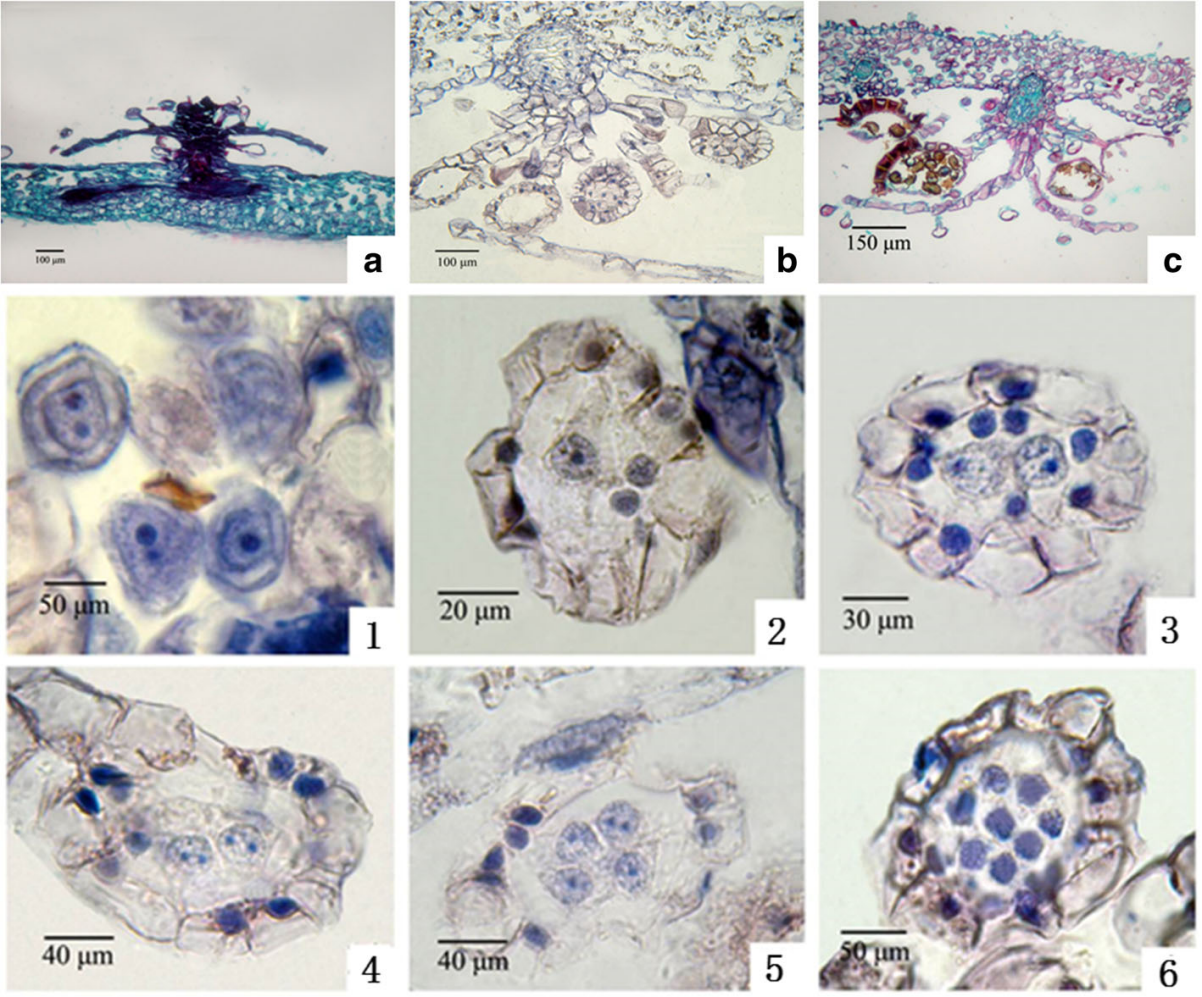
**a**, **b**, **c** are stage A, stage B, and stage C, respectively. 1 to 6 were different sporangial developmental phases in stage A.1: Enclosing sporangial initials in parietal cells, mononuclear and binuclear sporangial initials can be seen.2: Sporangial initial divided into outer tapetum cells and inner spore mother cells.3: The spore mother cells undergo the first meiosis.4: The two cells changed from mononuclear to binuclear.5: The two-cell binucleate divided into four-cells. At this time, the tapetum cells were darker and still visible.6: The four-cells divide into 8 cells and tapetum cells disintegrate gradually

Paraffin sections of the three stages of *D. fragrans* sporangia (including sporangiophore and indusium) were obtained (Fig. 13).

From the paraffin sections, stage B and stage C have similar cell numbers of sporangia, sporangiophore and indusium. Compared with B period, the tapetal cells disappeared completely at stage C. Paraffin sections showed great differences in sporangia in stage A, and according to the order of development, six paraffin sections were made (Fig. 13).

Paraffin sections (Fig. 13) showed that the size of sporangia at stage B and C was similar, and except for the disappearance of tapetum cells, the number of cells in other structures had little change. By counting the number of cells in multiple paraffin sections (not yet contributed), we found that the number of sporangial cells in stage A was much smaller than that in stage B and C.

The paraffin sections showed that the sporangia of stage A could be subdivided into at least 6 more precise stages of development with the number of cells from less to more. In the six subdivisions, the number of parietal cells gradually increased (Fig. 13 1–6), outer tapetum cells produced by mitosis of sporangial initials cell continue to divide into tapetum (Fig. 13.2–5). Spore mother cells were found to be produced in the second subdivision stage (Fig. 13-2), and 8 cells were formed in the sixth subdivision stage by meiosis (Fig. 13-6).

## Discussion

### Global gene transcription in the *D. fragrans* sporangia

High-throughput sequencing is an effective approach for studying the different developmental stages of organs. In this study, the expression patterns of genes in *D. fragrans* sporangia in different stages were identified by transcript profiling and comparative transcriptome analysis. A total of 94,705 Unigenes from 79.81 GB clean data were obtained, and approximately 46.47% Unigenes (44,006 Unigenes) could be functionally annotated in at least one public protein database (NR, Swiss-Prot, KEGG, COG, KOG, GO, eggNOG and Pfam). Approximately 53.53% of Unigenes (50,699 Unigenes) remained for functional annotation. These Unigenes either matched an unknown function protein, or none of their homologues was found. These Unigenes may be special in *D. fragrans* sporangia as novel transcripts.

Heat map of all differentially expressed genes can help us clearly see what happens to each stage in future research. It is worth pointing out that some poorly repeated data should not be abandoned, but more in-depth study. So far, we speculate that certain DEGs expression levels were not susceptible to external influences, and they regulate the basic development of the spores. Certain DEGs were susceptible to outside influence (such as group1, group.34, and group 66), and these DEGs may involve many resistance genes and metabolic pathways of some known/unknown;, and we will research a number of these DEGs in the future.

It is worth noting that Stage A has many different precise stages in sporangia, and transcription would vary by the phases. In this case, each precise stage specific expression gene may be averaged by different levels of expression at other precise stages.

### Multiple database analysis reveals possible core and family/species-specific features of the sporangia developmental process

Through comparative analysis of transcriptome data, we observed several characteristics of wild *D. fragrans* sporangia development at the transcriptome level as the core and family/species-specific features in *D. fragrans* or ferns.

First, secondary metabolism and plant hormone signal transduction were the main enrichment pathways between stage B and C. There were 6 KEGG pathways that were reliable at a corrected *P*-value less than 0.05 between stages B and C, and they were phenylpropanoid biosynthesis (4.486E-10), phenylalanine metabolism (6.370E-06), stilbenoid, diarylheptanoid and gingerol biosynthesis (0.005343), pentose and glucuronate interconversions (0.005863), cutin, suberin and wax biosynthesis (0.04584) and plant hormone signal transduction (0.04936). In addition to cutin, suberin and wax biosynthesis and glucuronate interconversions pathways, the other pathways seem to be related to plant stress resistance. We examined the detailed data in the heatmap (Additional file 4) and observed that the stress resistance-related GO term underwent dramatic changes during the BC period (most of which were up-regulated). These changes in gene expression may indicate a number of unknown plant stress resistance strategies.

Further research into these mechanisms will not only help us understand how ferns adapt to the environment but also may provide new ideas for crop improvement related to fruit and flowers.

Second, there were 660 DEGs between stage A and the B, and we observed that functional annotation and enrichment analyses of DGEs showed there were significant differences mainly associated with DNA replication and homologous recombination during this period. With further examination, we observed that all of these Unigenes in the two KEGG pathways were down-regulated. For instance, c116208.graph_c0, c109434.graph_c0, c114931.graph_c0, c116571.graph_c0, c117358.graph, c0, c101985.graph_c0 (Additional File 8) were matched to MCM 2, MCM 3, MCM 4, MCM5, MCM6 and MCM 7, respectively. The minichromosome maintenance proteins (MCMs) are highly conserved proteins widely observed from ancient creatures to higher eukaryotes and involved in the initiation of eukaryotic genome replication. MCM2~ 7 are the components of the minichromosome maintenance (MCM) complex, which acts as a DNA helicase during replication and elongation of DNA. There is a positive correlation between the overall level of MCM and cell proliferation and growth capacity. Therefore, the level of MCM also represents the different cell proliferation states [7–9].

Transcriptome data showed that MCM 2–7 declined significantly during stage B compared to stage A. This finding may mean that the frequency of cell division in stage A is significantly higher than that in stage B. It can be seen that the change of DNA replication related unigenes were consistent with the morphological observation (Fig. 13 1–6).

RAD51 and RAD54 (Additional File 8) were putative Unigenes for c110186.graph_c0 and c114077.graph_c0, respectively. Both of these Unigenes are important in meiotic recombination [10, 11]. In the model plant *Arabidopsis thaliana,* RAD51 has higher expression in reproductive tissues than other tissues and exhibits the highest expression level in young flower buds. At a cellular level, RAD 51 has lower levels of expression in flower primordia, higher levels in young anthers, highest levels in both female meiocytes and male meiocytes and is not detected in gametopjytes [10, 12, 13].

RAD54 has the highest levels of expression in flower buds and is present in flower buds at the protein level in *Arabidopsis thaliana* [11]. Both RAD51 and RAD54 showed very pronounced down regulation during the A to B stage (dropped by approximately 19 times and 16 times, respectively). qRT-PCR also showed the same changes (dropped by approximately 75 times and 16 times, respectively) (Additional File 8).

Based on the changes in DEG expression related to DNA replication, we speculated that there was a higher mitosis frequency in stage A than stage B and C. RAD51 and RAD54 were associated with homologous recombination and usually occurred at the meiosis stage of spore mother cells. These changes were consistent with our paraffin sections (Fig. 13).

Thirdly, we observed that the enrichment factor of cutin, suberin and wax biosynthesis pathway was in the top two (A vs B 1st 18.86, A vs C 2nd 4.34, B vs C 2nd 4.16) with a corrected *P*-value < 0.05 (0.000325797, 0.004360539, 0.045844412). To our surprise, there were only 10 Unigenes involved altogether. The putative gene for the 8 Unigenes (c108785.graph_c0, c121145.graph_c1, c123567.graph_c0, c69773.graph_c0, c98782.graph_c0, c112122.graph_c0, c112135.graph_c0, c117264.graph_c0) were omega-hydroxypalmitate O-feruloyl transferase. The other two putative genes were peroxygenase (c111664.graph_c0) and fatty acyl-CoA reductase (c120866.graph_c0).

All 8 omega-hydroxypalmitate O-feruloyl transferase-related Unigenes declined significantly over the three periods (Additional File 8). This putative gene is involved in the synthesis of aromatics of the suberin polymer and specifically affects the accumulation of the ferulate constituent of suberin in seeds. It is interesting that this gene was detected at the protein level in seed coats of *Arabidopsis* [14, 15]. Suberin is a polyester polymer in the cell walls of terrestrial plants, which protects plants from infection and other environmental pressures by controlling the transport of water and nutrients. This change may be related to the early development of sporangial stress resistance mechanism or the common characteristic of reproductive organ development of ferns and flowering plants, which is worthy of further study in the future.

Peroxygenase may be involved in the interaction between oil bodies and vacuoles during seed germination in spermatophytes. There is no visible phenotype after gene ablation but retarded growth during the first 48 h after germination. As described in literatures, peroxygenase was specific expressed in seeds and restricted to the developing embryo where its transcription was enhanced in protoderm and vasculature during the cotyledon stage of seed maturation and decreased gradually after germination [16–19]. Fatty acyl-CoA reductase 2 was involved in the synthesis of the lipid component in sporopollenin and expressed in the tapetum [20, 21].

As we can see, the expression pattern of DEGs in cutin, suberin and wax biosynthesis during the development of sporangia was consistent with that in the flowering to seed development process of flowering plants. From an evolutionary point of view, flowering plants appear after ferns. Whether their reproductive organs have similar manifestations is worth further study. Paraffin sections showed that tapetal cells and spore mother cells were produced simultaneously (Fig. 13.2), gradually collapsed when spore mother cells split into eight cells (Fig. 13.6), and disappeared completely at stage C. This finding is in accordance with the changes in the expression of c120866.graph_c0.

### Discussion of genes related to the reproductive organs of flowering plants

A large number of DEGs were annotated to transcription factors associated with floral and seed development.

MADS-box genes play very important roles in the process of plant development, and we observed 3 putative DEGs annotated to known genes related to the seed and gametophyte development peak at stage C (c109839.graph_c1, c116857.graph_c0, c93709.graph_c0, and the putative genes were AGL62, AGL62, AGL61, respectively) (Additional File 8). AGL62 expressed in the endosperm but not in the embryo during syncytial endosperm development, and plants lacking AGL62 show a seed-lethal phenotype with premature endosperm cellularization [22]. AGL61 controls central cell differentiation during female gametophyte development and is expressed in the final stages of embryo sac development [23, 24].

In the NAC family, c117598.graph_c0 (putative gene NAC transcription factor ONAC010) is associated with male fertility. Reduced expression of NAC5 via RNAi leads to male sterility [25]. c117039.graph_c0 (putative gene B3 domain-containing transcription factor ABI3) (Additional File 8) participates in abscisic acid-regulated gene expression during seed development. This gene regulates the transcription of SGR1 and SGR2, which are involved in leaf and embryo degreening [26, 27]. These characteristics imply that there may be similarity between the sporangia development in stage C and the development of plant seeds.

We noted that there were few Unigene expression peaks in stage B, and c92612.graph_c0 putative gene TFL1) (Additional File 8) was an exception. This gene was the Unigene with the highest expression in stage B. TFL1 not only controls inflorescence meristem identity, required for maintenance of an indeterminate inflorescence, but also plays a role in the regulation of the time of flowering in the long-day flowering pathway [28, 29]. The significance of such high levels of expression in sporangia is worth studying in the in future.

## Conclusions

Using *D. fragrans* sporangia from nine samples of three periods, 79.81 Gb Clean data were obtained (a sample of not less than 7.95GB, Q30 = 90.72%). After assembly and data analysis, 94,705 Unigenes were obtained, including 27,275 Unigenes with lengths over 1 KB, and 44,006 Unigenes were annotated in the database. In all, 10,584 SSR markers and 349,885 SNPs were obtained through data analysis. Eight Unigenes were verified by qRT-PCR, and the results were consistent with transcriptome data.

In stage A, DEGs associated with DNA replication and homologous recombination suggest that plants may undergo frequent mitosis and meiosis at this time. In stage AB and stage BC, the most reliable pathway of KEGG enrichment of DEGs were dominated by secondary metabolic pathways. Several genes associated with floral and seed development may also perform biological functions in sporangium.

This study provides a rare database resource for conducting thorough multilevel research for sporangia of *D. fragrans* and other ferns.

## Additional files

Additional File 1: Software list and nucleic acid coding table (PDF 220 kb)

Additional File 2: Thirty-six scatterplot diagrams of gene pairwise correlation (RAR 1443 kb)

Additional File 3: All DEGs list (XLSX 1713 kb)

Additional File 4: Heatmap of enrichment of GO term with 66 groups (PDF 3453 kb)

Additional File 5: KEGG enrichment results (XLSX 46 kb)

Additional File 6: Unigenes, ORF, FPKM, SNPs and primers of 8 selected genes (XLSX 48 kb)

Additional File 7: Relative expression level of 8 selected genes (XLSX 70 kb)

Additional File 8: Expression and relative expression of Unigenes involved in this article (PDF 440 kb)

## Abbreviations

COG: Clusters of Orthologous Groups
FPKM: Fragment per kilobase per million mapped reads
GO: Gene Ontology
KEGG: Kyoto Encyclopedia of Genes and Genomes
KOG: euKaryotic Orthologous Groups
MCMs: minichromosome maintenance proteins
NCBI: National center for biotechnology information
Nr: NCBI non-redundant protein sequences
Pfam: Protein family
Swiss- Prot: Swiss institute of bioinformatics

## References

[1] Masuyama S (2008). Cryptic species in the fern *Ceratopteris thalictroides* (Parkeriaceae). III. Referential diagnostic characters of three cryptic species. J Plant Res.

[2] Noblin X, Rojas NO, Westbrook J, Llorens C, Argentina M, Dumais J. The Fern Sporangium: A Unique Catapult. Science (80-.). 2012;335:1322–1322 10.1126/science.1215985 22422975

[3] Vasco A, Smalls TL, Graham SW, Cooper ED, Wong GK, Stevenson DW (2016). Challenging the paradigms of leaf evolution: Class III HD-Zips in ferns and lycophytes. New Phytol.

[4] Poppinga S, Haushahn T, Warnke M, Masselter T, Speck T (2015). Sporangium exposure and spore release in the peruvian maidenhair fern (Adiantum peruvianum, Pteridaceae). PLoS One.

[5] Zhang L-B, Zhang L, Dong S-Y, Sessa EB, Gao X-F, Ebihara A (2012). Molecular circumscription and major evolutionary lineages of the fern genus Dryopteris (Dryopteridaceae). BMC Evol Biol.

[6] Grabherr MG, Haas BJ, Yassour M, Levin JZ, Thompson DA, Amit I (2011). Full-length transcriptome assembly from RNA-Seq data without a reference genome. Nat Biotechnol.

[7] Masai H, Matsumoto S, You Z, Yoshizawa-Sugata N, Oda M (2010). Eukaryotic Chromosome DNA Replication: Where, When, and How?. Annu Rev Biochem.

[8] Schwacha A, Bell SP (2001). Interactions between two catalytically distinct MCM subgroups are essential for coordinated ATP hydrolysis and DNA replication. Mol Cell.

[9] Kusunoki S, Ishimi Y (2014). Interaction of human minichromosome maintenance protein-binding protein with minichromosome maintenance 2–7. FEBS J.

[10] Li W, Chen C, Markmann-Mulisch U, Timofejeva L, Schmelzer E, Ma H, et al. The Arabidopsis AtRAD51 gene is dispensable for vegetative development but required for meiosis. Proc Natl Acad Sci U S A. 2004;101:10596–601. 10.1073/pnas.0404110101 PMC489980 15249667

[11] Osakabe K, Abe K, Yoshioka T, Osakabe Y, Todoriki S, Ichikawa H (2006). Isolation and characterization of the RAD54 gene from *Arabidopsis thaliana*. Plant J.

[12] Doutriaux MP, Couteau F, Bergounioux C, White C (1998). Isolation and characterisation of the RAD51 and DMC1 homologs from *Arabidopsis thaliana*. Mol Gen Genet.

[13] Osakabe K, Yoshioka T, Ichikawa H, Toki S (2002). Molecular cloning and characterization of RAD51-like genes from *Arabidopsis thaliana*. Plant Mol Biol.

[14] Molina I, Li-Beisson Y, Beisson F, Ohlrogge JB, Pollard M (2009). Identification of an Arabidopsis Feruloyl-Coenzyme A Transferase Required for Suberin Synthesis. Plant Physiol.

[15] Gou J-Y, Yu X-H, Liu C-J (2009). A hydroxycinnamoyltransferase responsible for synthesizing suberin aromatics in Arabidopsis. Proc Natl Acad Sci.

[16] Poxleitner M, Rogers SW, Lacey Samuels A, Browse J, Rogers JC (2006). A role for caleosin in degradation of oil-body storage lipid during seed germination. Plant J.

[17] Chen JC, Tsai CC, Tzen JT (1999). Cloning and secondary structure analysis of caleosin, a unique calcium-binding protein in oil bodies of plant seeds. Plant Cell Physiol.

[18] Hsiao ES, Tzen JT (2011). Ubiquitination of oleosin-H and caleosin in sesame oil bodies after seed germination. Plant Physiol Biochem Ppb.

[19] Nuccio ML TT. ATS1 and ATS3 two novel embryo-specific genes in Arabidopsis thaliana. Plant Molecular Biology. 1999;39(6):1153–63. 10.1023/a:1006101404867 10380802

[20] Doan TT, Carlsson AS, Hamberg M, Bülow L, Stymne S, Olsson P (2009). Functional expression of five Arabidopsis fatty acyl-CoA reductase genes in *Escherichia coli*. J Plant Physiol.

[21] Aarts MGM, Hodge R, Kalantidis K, Florack D, Wilson ZA, Mulligan BJ (1997). The Arabidopsis MALE STERILITY 2 protein shares similarity with reductases in elongation/condensation complexes. Plant J.

[22] Kang I-H, Steffen JG, Portereiko MF, Lloyd A, Drews GN (2008). The AGL62 MADS Domain Protein Regulates Cellularization during Endosperm Development in Arabidopsis. Plant Cell Online.

[23] Steffen JG, Kang I-H, Portereiko MF, Lloyd A, Drews GN (2008). AGL61 Interacts with AGL80 and Is Required for Central Cell Development in Arabidopsis. Plant Physiol.

[24] Bemer M, Wolters-Arts M, Grossniklaus U, Angenent GC (2008). The MADS Domain Protein DIANA Acts Together with AGAMOUS-LIKE80 to Specify the Central Cell in Arabidopsis Ovules. Plant Cell Online.

[25] Distelfeld A, Pearce SP, Avni R, Scherer B, Uauy C, Piston F (2012). Divergent functions of orthologous NAC transcription factors in wheat and rice. Plant Mol Biol.

[26] Alonso R, Onate-Sanchez L, Weltmeier F, Ehlert A, Diaz I, Dietrich K (2009). A Pivotal Role of the Basic Leucine Zipper Transcription Factor bZIP53 in the Regulation of Arabidopsis Seed Maturation Gene Expression Based on Heterodimerization and Protein Complex Formation. Plant Cell Online.

[27] Delmas F, Sankaranarayanan S, Deb S, Widdup E, Bournonville C, Bollier N (2013). ABI3 controls embryo degreening through Mendel’s I locus. Proc Natl Acad Sci.

[28] Bradley D (1997). Inflorescence Commitment and Architecture in Arabidopsis. Science (80-.).

[29] Ratcliffe OJ, Amaya I, Vincent CA, Rothstein S, Carpenter R, Coen ES (1998). A common mechanism controls the life cycle and architecture of plants. Development.

