# Supplementary material for: Global transcriptome analysis and characterization of Dryopteris fragrans (L.) Schott sporangium in different developmental stages
Source: BMC Genomics. 2018 Jun 18;19:471. doi: 10.1186/s12864-018-4843-2 (PMC6006573; doi:10.1186/s12864-018-4843-2)
Supplement: Supplementary file 1 — Software list and nucleic acid coding table (PDF 220 kb) [file 12864_2018_4843_MOESM1_ESM.pdf]

Software list

Tools	Description	Linkages
Trinity	A Full-length transcriptome assembly tool from RNA-Seq data without a reference genome	https://github.com/trinityrnaseq/trinityrnaseq/wiki
TransDecoder	An tool for identifying candidate coding regions within transcript sequences	http://sourceforge.net/projects/transdecoder/
MISA	A MIcroSAtellite identification tool	http://pgrc.ipk-gatersleben.de/misa/misa.html
BLAST	Basic Local Alignment Search Tool	http://blast.ncbi.nlm.nih.gov/Blast.cgi
KOBAS2.0	A software to identify statistically significantly enriched pathways using hypergeometric test	http://kobas.cbi.pku.edu.cn/help.do
HMMER	A tool for searching sequence databases for sequence homologs, and for making sequence alignments	http://hmmer.org/
RSEM	An accurate tool for quantifying transcript abundances from RNA-Seq data	http://deweylab.github.io/RSEM/
STAR	An ultrafast universal RNA-seq aligner	https://github.com/alexdobin/STAR
GATK	A wide variety of tools with a primary focus on variant discovery and genotyping	https://software.broadinstitute.org/gatk/
EBSeq	An R package for RNA-Seq Differential Expression Analysis based on Bayesian approach	https://www.biostat.wisc.edu/~kendzior/EBSEQ/
topGO	An R package for gene ontology enrichment analysis	http://www.bioconductor.org/packages/release/bioc/html/topGO.html

Nucleic acid coding table

Nucleic Acid Code	Meaning	Mnemonic
A	A	Adenine
C	C	Cytosine
G	G	Guanine
T	T	Thymine
U	U	Uracil
R	A or G	puRine
Y	C, T or U	pYrimidines
K	G, T or U	bases which are Ketones
M	A or C	bases with aMino groups
S	C or G	Strong interaction
W	A, T or U	Weak interaction
B	not A (i.e. C, G, T or U)	Bcomes after A
