# Supplementary figures and images for: Global transcriptome analysis and characterization of Dryopteris fragrans (L.) Schott sporangium in different developmental stages
Source: BMC Genomics. 2018 Jun 18;19:471. doi: 10.1186/s12864-018-4843-2 (PMC6006573; doi:10.1186/s12864-018-4843-2)

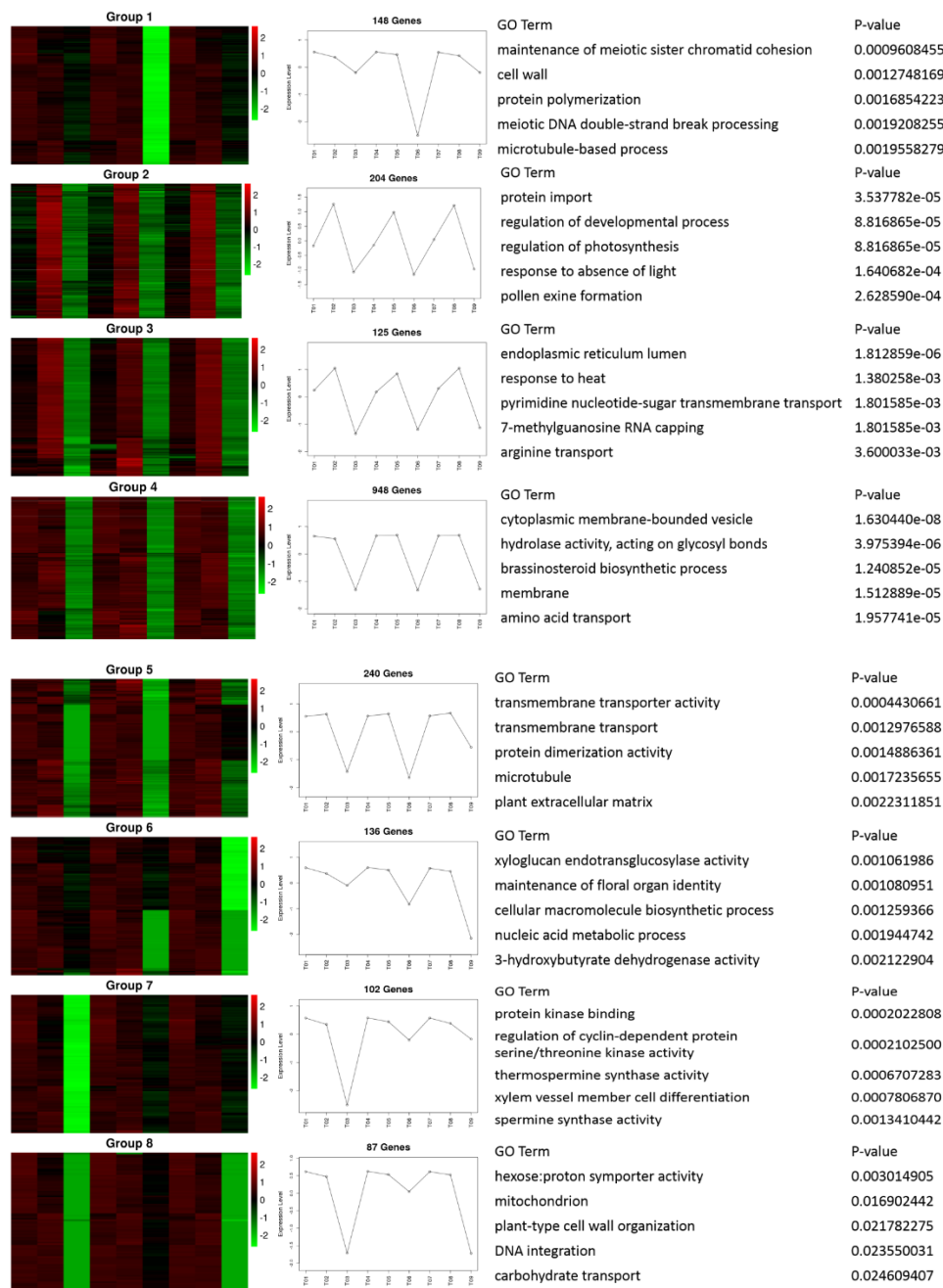

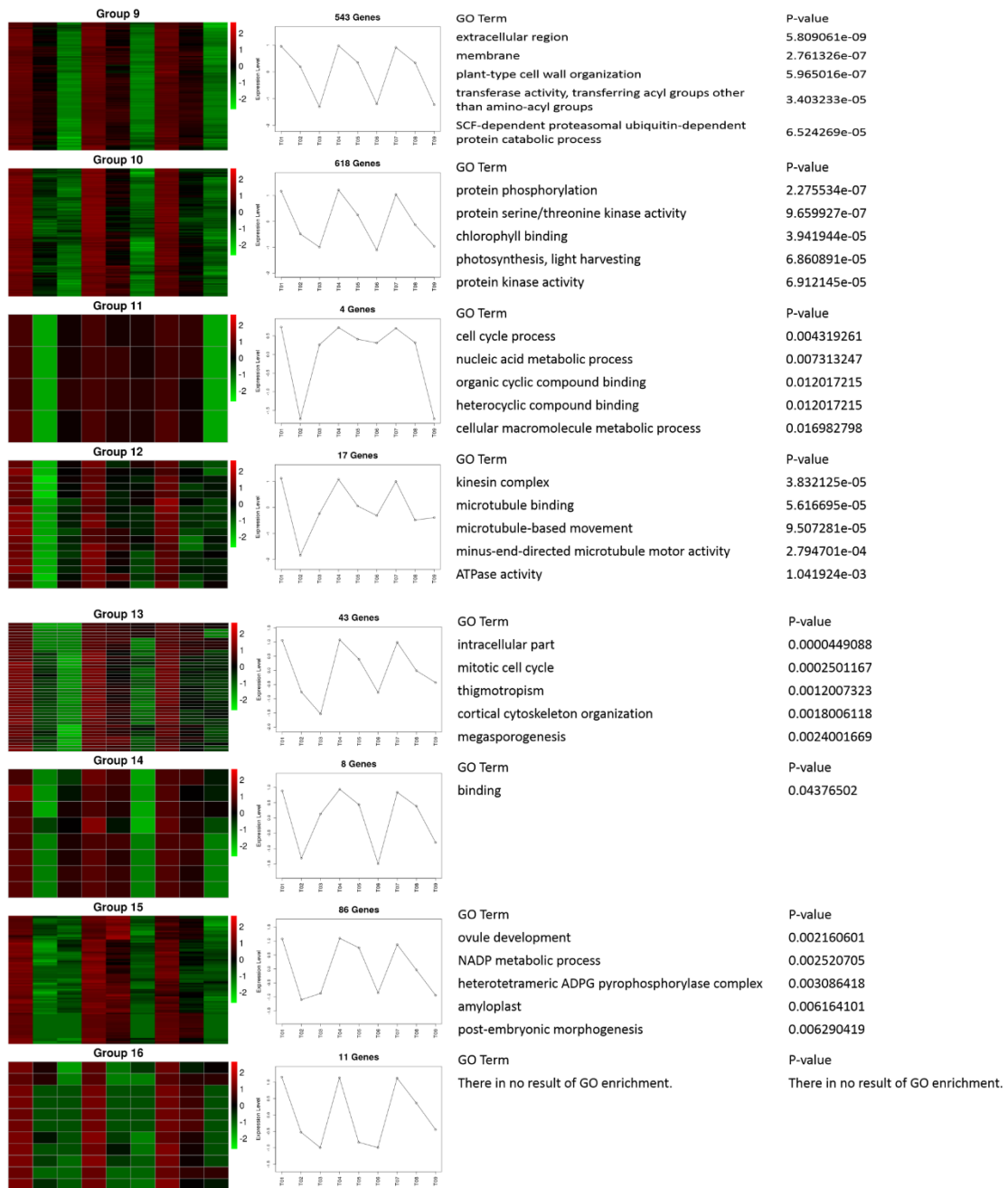

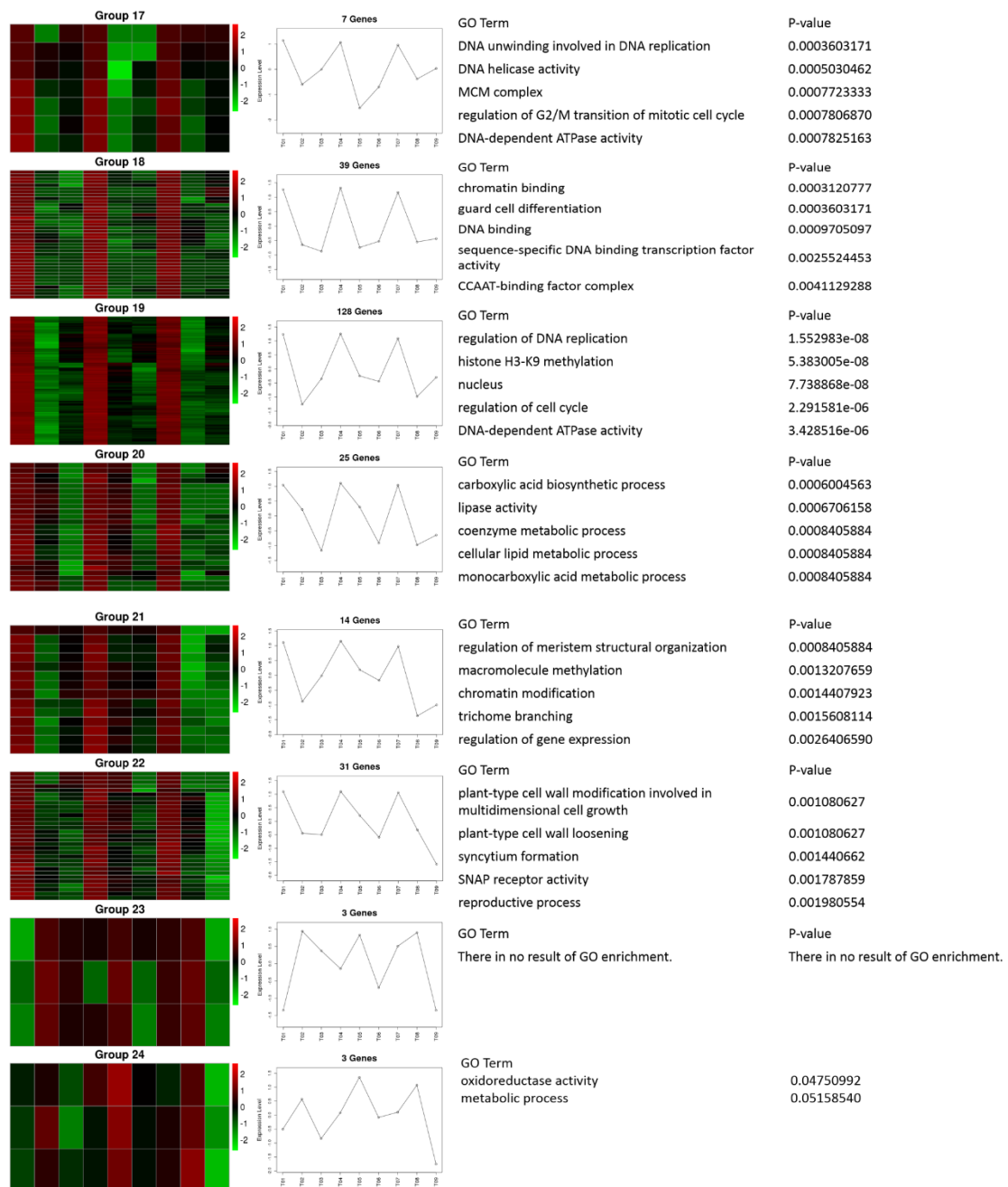

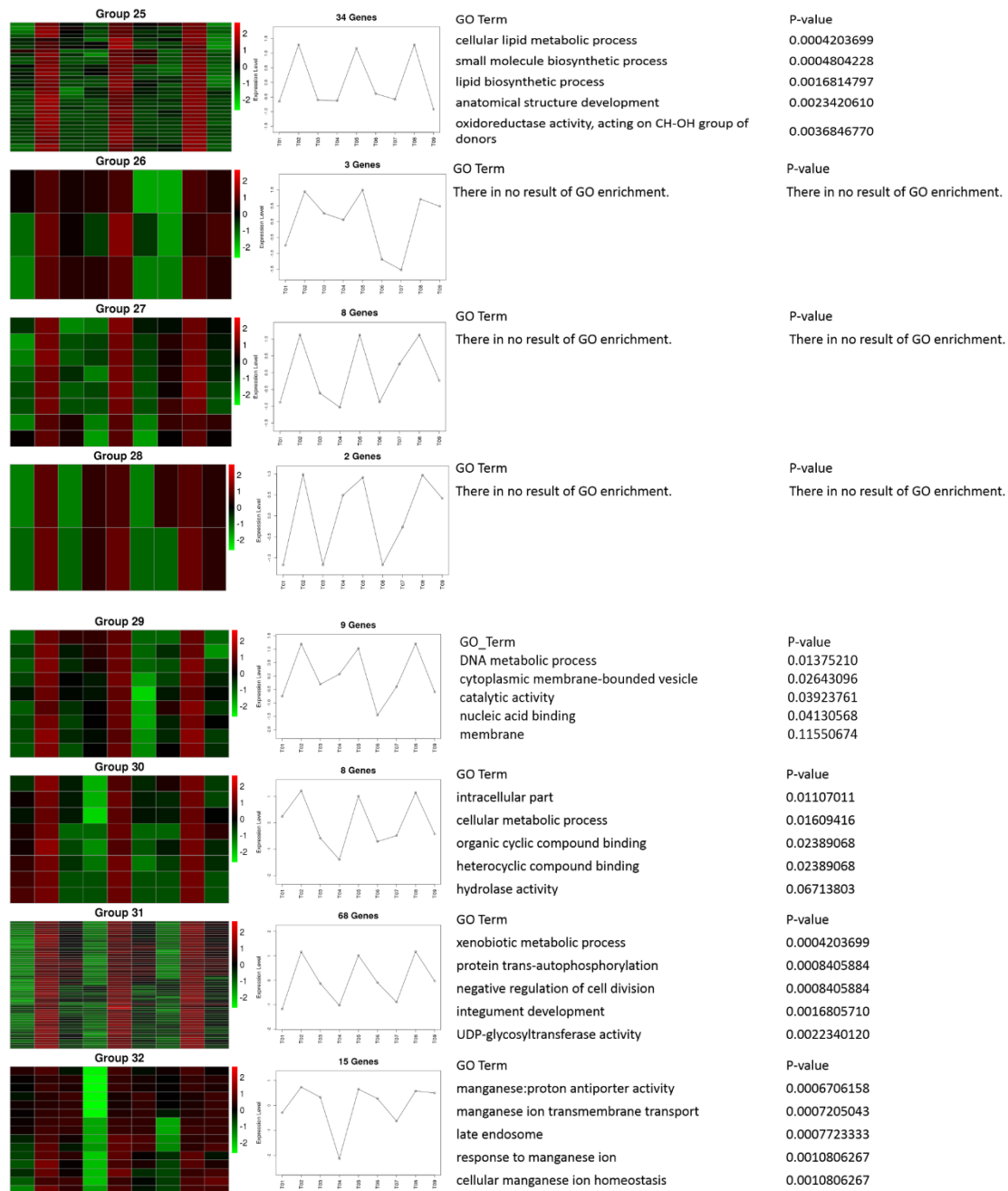

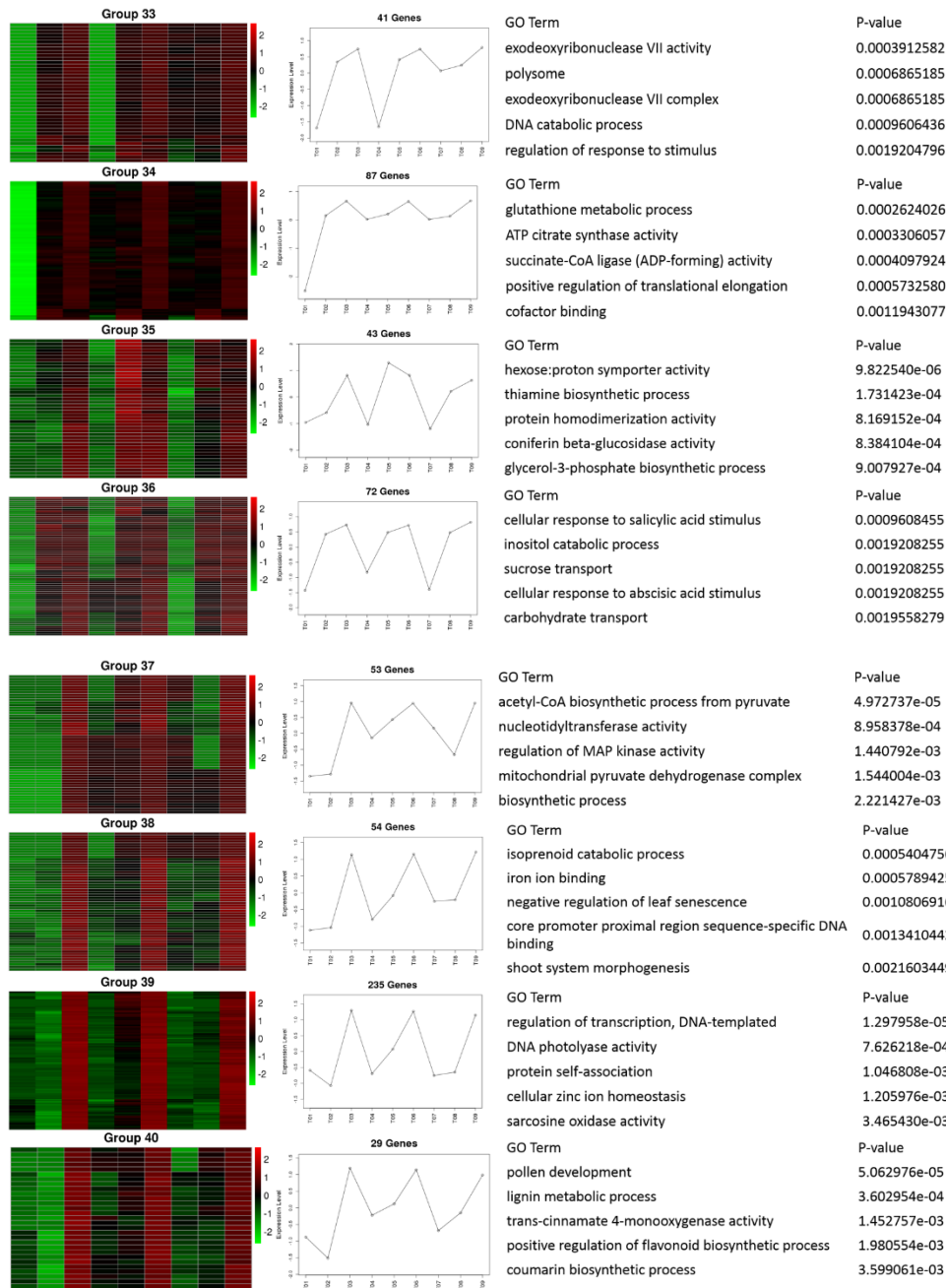

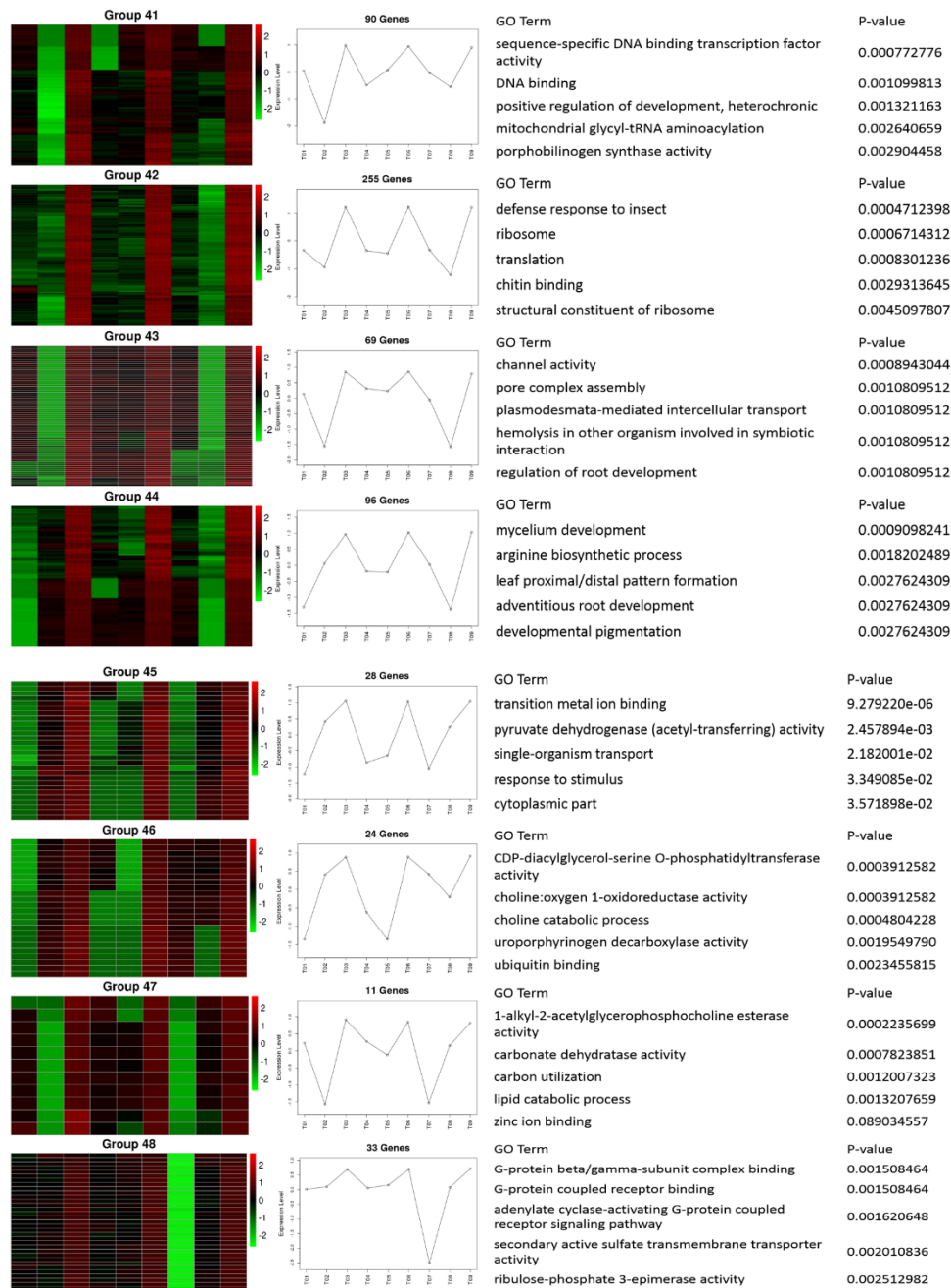

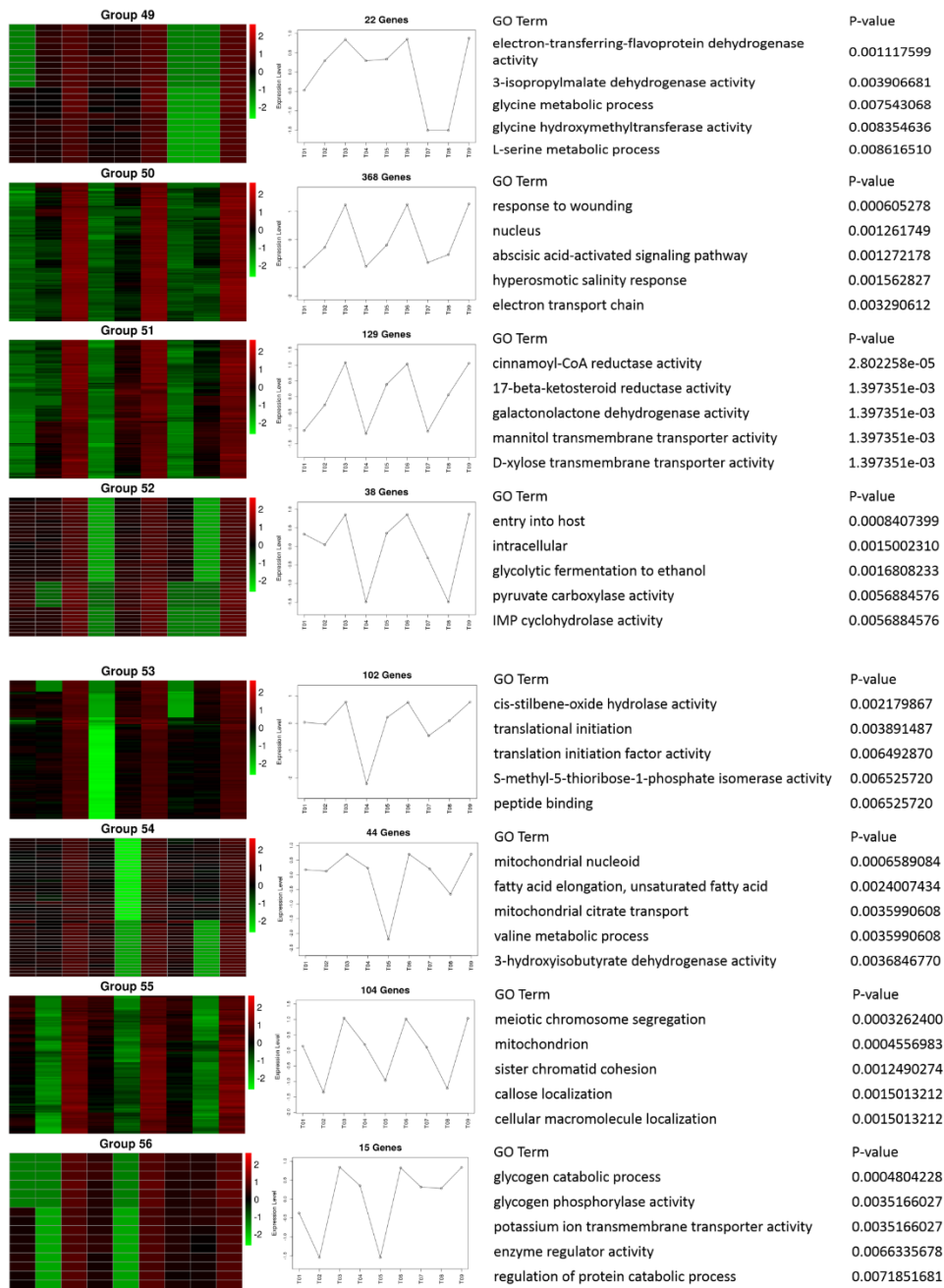

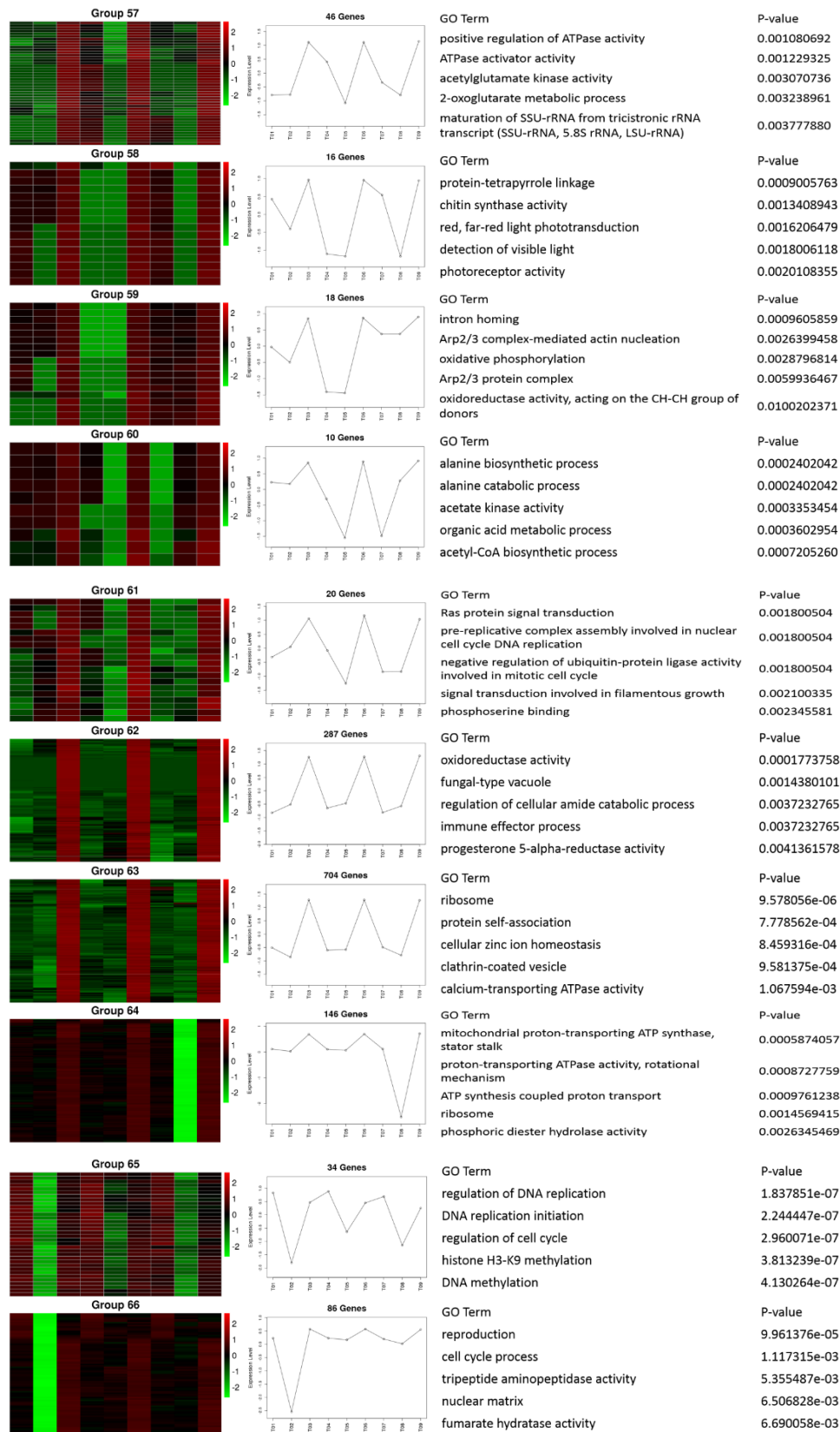

Supplement: Supplementary file 4 — Heatmap of enrichment of GO term with 66 groups (PDF 3453 kb) [file 12864_2018_4843_MOESM4_ESM.pdf]
