# Supplementary material for: Global transcriptome analysis and characterization of Dryopteris fragrans (L.) Schott sporangium in different developmental stages
Source: BMC Genomics. 2018 Jun 18;19:471. doi: 10.1186/s12864-018-4843-2 (PMC6006573; doi:10.1186/s12864-018-4843-2)

Expression & relative expression of unigenes in involved in this article, all data can be found from the relevant original file.

* representing data based on 2 biological repeats

c114077.graph_c0

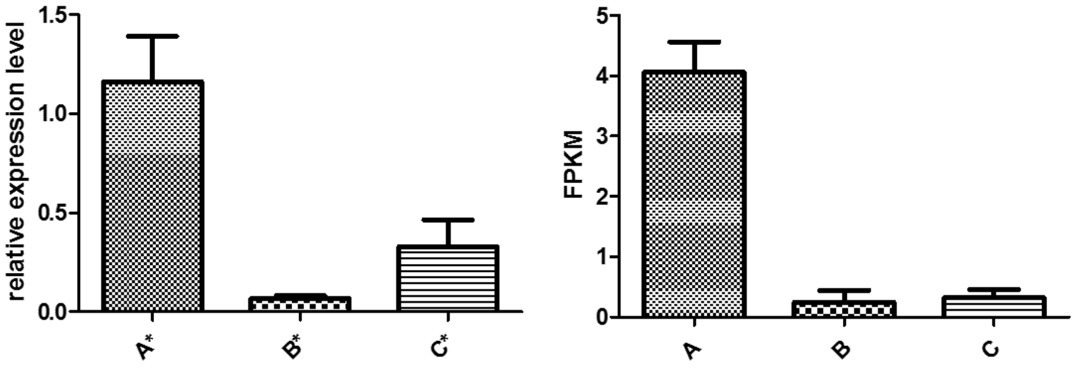

c110186.graph_c0

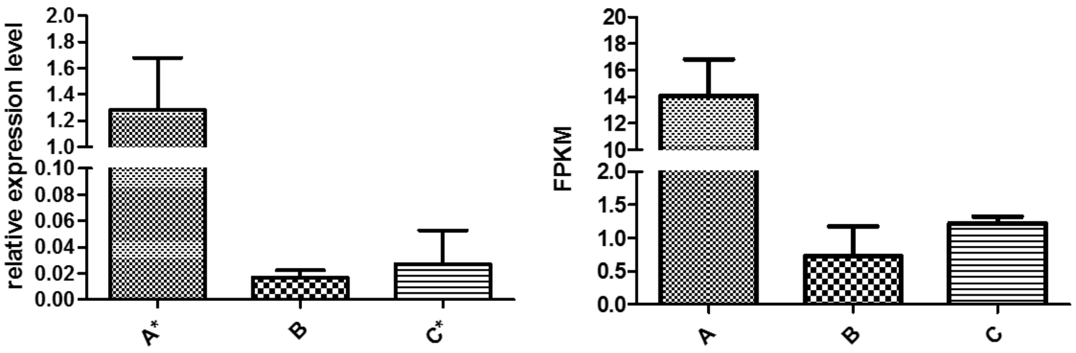

c111664.graph_c0

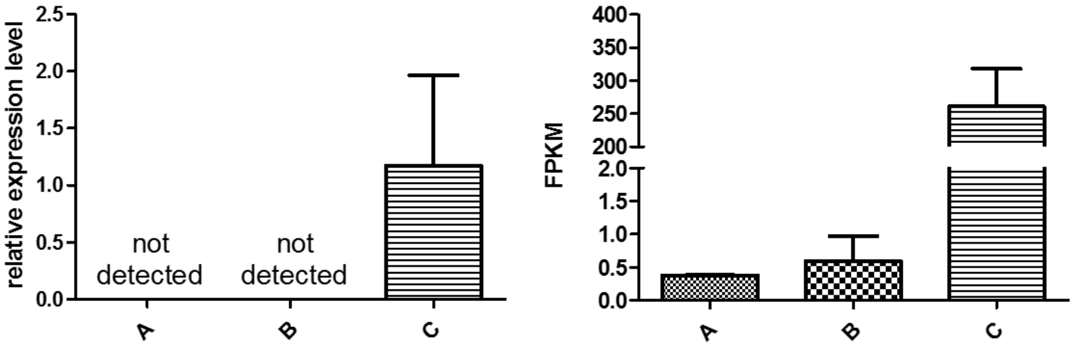

c120866.graph_c0

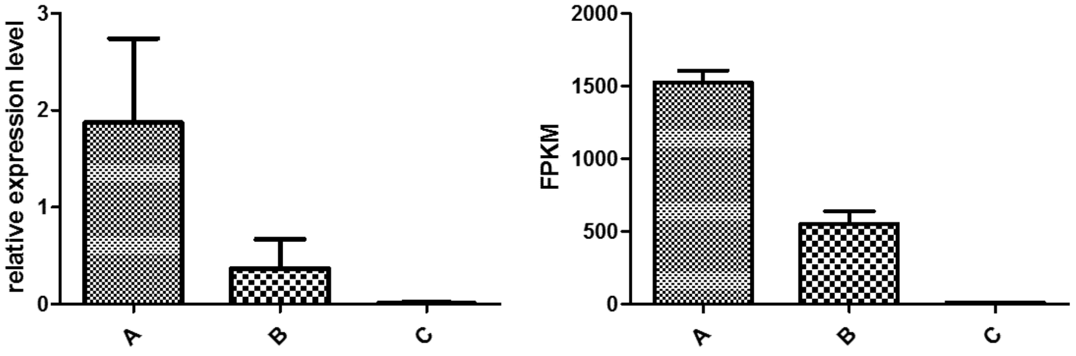

c121145.graph_c1

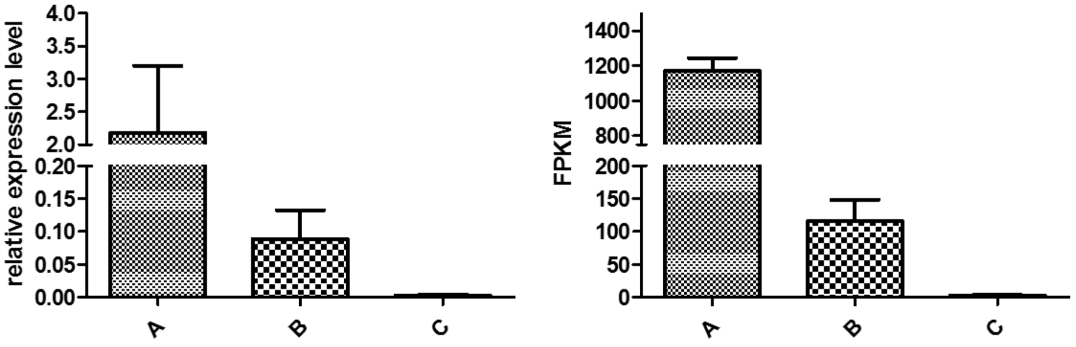

c109839.graph_c1

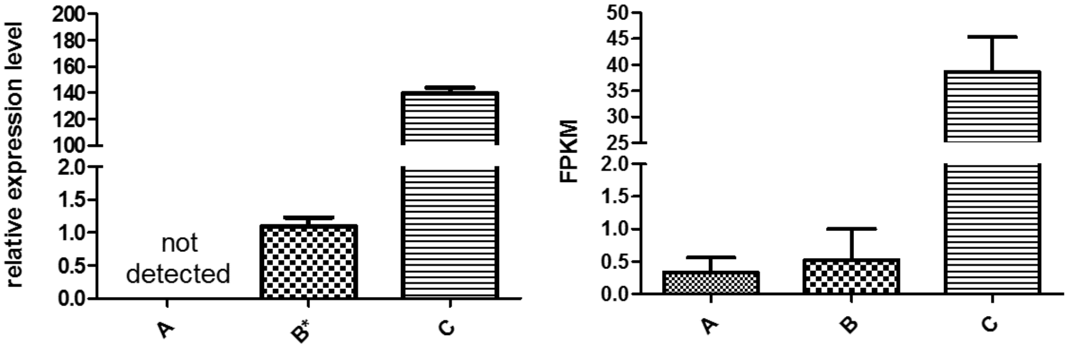

c117958.graph_c0

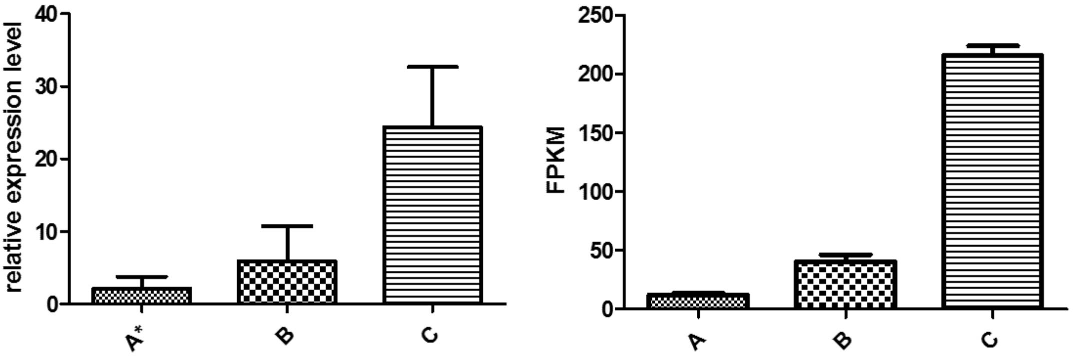

c92612.graph_c0

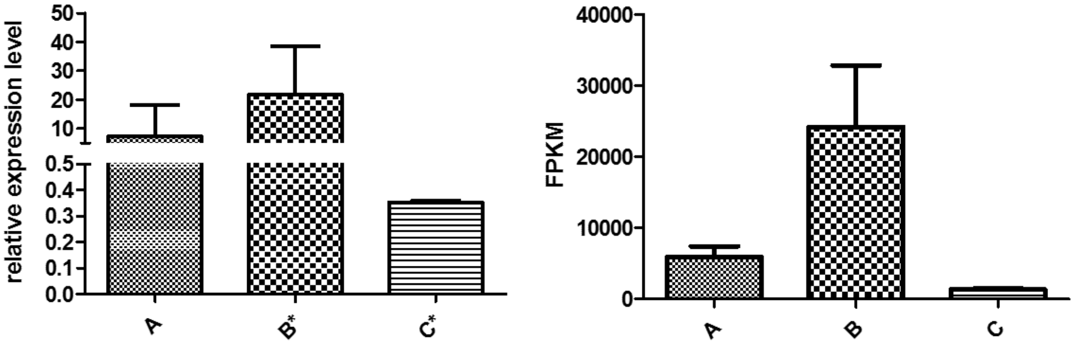

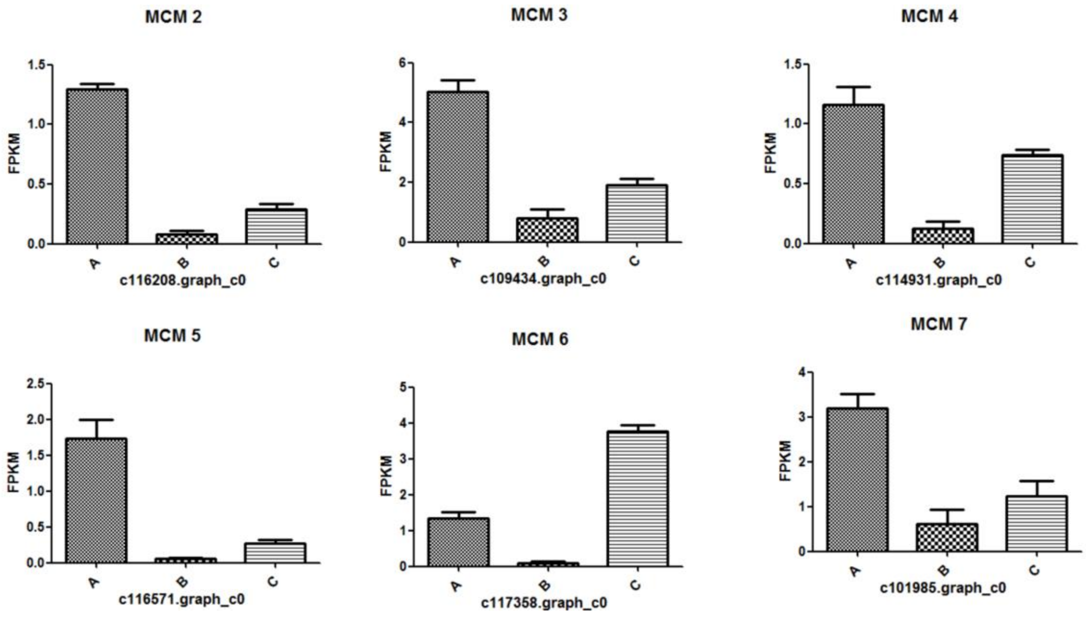

Putative genes belong Omega-hydroxypalmitate O-feruloyl transferase

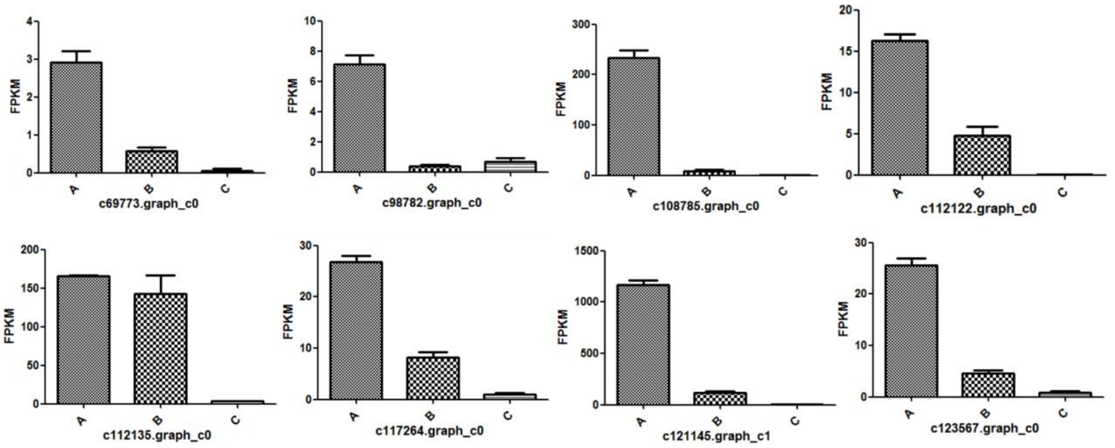

AGL62

c109839.graph_c1

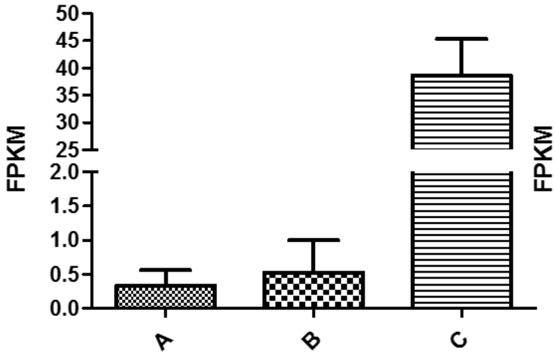

c116857.graph_c0

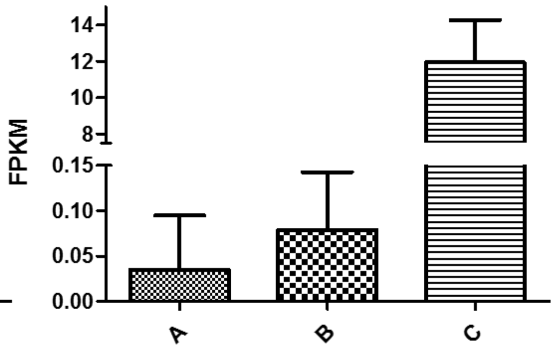

AGL61

c93709.graph_c0

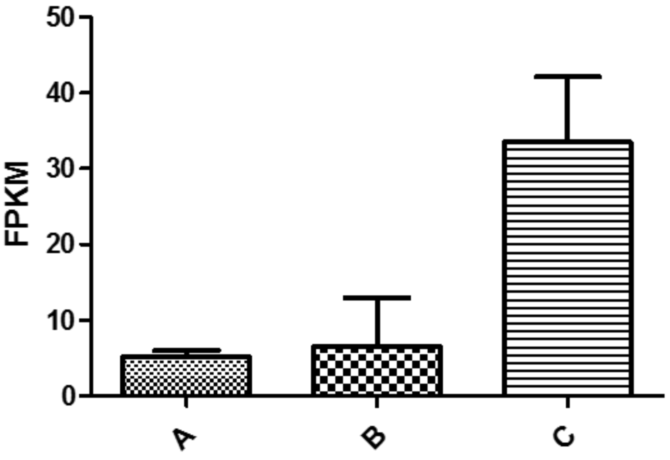

B3 domain-containing transcription factor ABI3

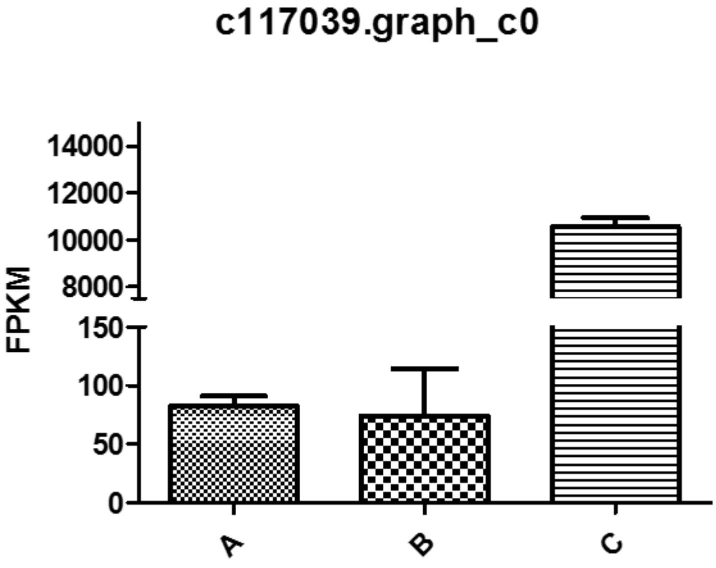

Supplement: Supplementary file 8 — Expression and relative expression of Unigenes involved in this article (PDF 440 kb) [file 12864_2018_4843_MOESM8_ESM.pdf]
